# Soil microbial community assembly and stability are associated with potato (*Solanum tuberosum* L.) fitness under continuous cropping regime

**DOI:** 10.3389/fpls.2022.1000045

**Published:** 2022-10-03

**Authors:** Songsong Gu, Xingyao Xiong, Lin Tan, Ye Deng, Xiongfeng Du, Xingxing Yang, Qiulong Hu

**Affiliations:** ^1^ Hunan Agricultural University, Changsha, China; ^2^ Key Laboratory for Environmental Biotechnology, Research Center for Eco-Environmental Sciences, Chinese Academy of Sciences (CAS), Beijing, China; ^3^ Agricultural Genomics Institute at Shenzhen, Chinese Academy of Agricultural Sciences, Shenzhen, China; ^4^ Hunan Center of Crop Germplasm Resources and Breeding Crop, Changsha, China

**Keywords:** continuous cropping obstacles, community assembly, network stability and complexity, potato cultivar, bacteria and fungi

## Abstract

Continuous cropping obstacles caused by the over-cultivation of a single crop trigger soil degradation, yield reduction and the occurrence of plant disease. However, the relationships among stability, complexity and the assembly process of soil microbial community with continuous cropping obstacles remains unclear. In this study, molecular ecological networks analysis (MENs) and inter-domain ecological networks analysis (IDENs), and a new index named cohesion tools were used to calculate the stability and complexity of soil microbial communities from eight potato cultivars grown under a continuous cropping regime by using the high-throughput sequencing data. The results showed that the stability (*i.e.*, robustness index) of the bacterial and fungal communities for cultivar ZS5 was significantly higher, and that the complexity (*i.e.*, cohesion values) was also significantly higher in the bacterial, fungal and inter-domain communities (*i.e.*, bacterial-fungal community) of cultivar ZS5 than other cultivars. Network analysis also revealed that *Actinobacteria* and *Ascomycota* were the dominant phyla within intra-domain networks of continuous cropping potato soil communities, while the phyla *Proteobacteria* and *Ascomycota* dominated the correlation of the bacterial-fungal network. Infer community assembly mechanism by phylogenetic-bin-based null model analysis (iCAMP) tools were used to calculate the soil bacterial and fungal communities’ assembly processes of the eight potato cultivars under continuous cropping regime, and the results showed that the bacterial community was mainly dominated by deterministic processes (64.19% - 81.31%) while the fungal community was mainly dominated by stochastic processes (78.28% - 98.99%), indicating that the continuous-cropping regime mainly influenced the potato soil bacterial community assembly process. Moreover, cultivar ZS5 possessed a relatively lower homogeneous selection, and a higher TP, TN, AP and yield than other cultivars. Our results indicated that the soil microbial network stability and complexity, and community assemble might be associated with yield and soil properties, which would be helpful in the study for resistance to potato continuous cropping obstacles.

## Introduction

Soil degradation refers to a significant decrease in soil quality and productivity affected by a decline in soil fertility, deterioration of soil structure, excess salinity, soil erosion, desertification, acidification, nutrient loss, and chemical contamination (Chen et al., 2018). Generally, continuous cropping obstacles caused by over-cultivation have been considered to be one of the main types of soil degradation. Potato (*Solanum tuberosum* L.) has become the fourth major food crop after rice, maize and wheat in the world with an annual production of more than 300 million metric tons (Pfeiffer et al., 2017). However, due to the limited amount of cultivatable land, the blind pursuit of economic benefits and the lack of scientific planting concepts, a large proportion of potato cultivation uses a monoculture regime, resulting in continuous-cropping obstacle with unhealthy growth as well as decreases in yield and quality (Qin et al., 2017). For instance, continuous-cropping obstacle could lead to a decrease in the content of potassium, phosphorus and nitrogen in the soil, an imbalance in the utilization of plant nutrients, and it accelerates the accumulation of plant autotoxins (such as phenolic acid) and promotes the epidemic of plant diseases (Aparicio & Costa, 2007).

Plant-microbiome interactions influence a wide range of biogeochemical processes, including mineralization of organic matter (Fontaine et al., 2007; Hu et al., 2020) and the cycling of biologically critical elements such as potassium, nitrogen and carbon (Mendes et al., 2014). It is well established that the composition and relative abundance of soil microbiomes play essential roles in enhancing soil quality, improving soil ecosystem functions, and maintaining plant health and growth (Helgason et al., 2009; Kong et al., 2011). Soil contains a huge number of beneficial microorganisms that drive plant health and productivity (van der Heijden et al., 2008), and serves as protectants against phytopathogens (Philippot et al., 2013). Some research has shown that host cultivar (genotype) could have an important impact on the root-associated microbiota (Lundberg et al., 2012), and rhizosphere effects indicated that the microbial community structure in the rhizosphere often displayed significant differences between cultivars (Bokulich et al., 2014). At present, the contributions of potato cultivars in shaping the soil microbial community under continuous cropping regime is poorly understood (Qin et al., 2017).

A microbial community with higher stability and service functions could impose a positive impact, with improvement in the nutrient cycling of various organic and inorganic materials in soil ecosystems (Rogers & Tate, 2001). With the development of high-throughput sequencing, related data-mining technologies and tools for analyzing the diversity, composition and structure of soil microbial community have been greatly advanced (Donn et al., 2015), these include the molecular ecology network, an effective and feasible way to reveal the interactions among organisms in a wide range of environments (Krause et al., 2003). An ecological network analysis is usually built to reveal various biological interactions within an ecosystem, such as complicated positive (e.g., commensalism and mutualisms) and negative (e.g., predation and competition) interactions (Chow et al., 2014). Network analysis can identify keystone species or other important microorganisms that may have the greatest impact on microbial community structure and potential functions by identifying the most connected microbial populations or analyzing the distribution of nodes and linkages (Berry and Widder, 2014; Banerjee et al., 2016; Layeghifard et al., 2017). Furthermore, ecological network analysis also offers a powerful approach for evaluating ecosystem stability (Dunne et al., 2002a), by characterizing complicated ecological relationships among microbial taxa (Deng et al., 2012). However, the question remains open as to whether potato cultivars leave an imprint on the stability and complexity of soil microbial community networks.

The objectives of this study were to (i) investigate the differences of bacterial and fungal communities’ diversity and structure in the soil of different potato cultivars under continuous-cropping regime, (ii) evaluate the intra-domain and inter-domain networks stability and complexity among eight potato cultivar soils, and (iii) to reveal the bacterial and fungal communities’ assembly processes under continuous cropping regime. Our findings will provide data support and theoretical basis for improving microecological environment of soil under continuous potato cropping through the logical selection of potato cultivars.

## Material and methods

### Site description and sample collection

The study sites were situated in the Wangcheng district (28°20′51.288′′N, 112°49′10.376′′E) of Changsha city, Hunan province, China (**Figure S1**). The region experiences a warm temperate continental monsoon climate with annual average temperature of 15-17°C, annual average frost-free period of 270-280 days, annual average sunshine 1700-1800 hours and annual average rainfall of about 1300-1400 mm. The soil type was sandy loam soil.

Eight potato cultivars were cultivated in their respective experimental plots under a continuous cropping regime since 2013, with each plot size being larger than 75 m^2^. These potato experimental plots had similar environmental characteristics, such as altitude, slope position and slope aspect, and similar agronomic management. They included the potato cultivars Dongnong303 (DN303), E14(E14), Huacai1 (HC1), Huaen1 (HE1), Huashu4 (HS4), Xingjia2 (XJ2), Zhongshu5 (ZS5), and Zhongshu4 (ZS4), all of which are popular cultivars in China. Huaen1 (HE1-R) and Xingjia2 (XJ2-R) were planted under a rice-potato rotation regime, which were used for the analysis and comparison of two planting regimes.

In each experimental plot, we randomly selected six rows of potato plants, and collected the bulk soil samples (0-20 cm soil layer) from six random points within each row and mixed them as a composite sample. In total, 48 soil composite samples were collected in April 2017. The samples were divided into two parts, one part was used to determine the physicochemical properties while the other part was sieved to 2.0 mm and stored at -80°C for use in molecular experiments (DNA extraction).

### Soil physicochemical analyses

For each soil sample, the contents of total phosphorus (TP), total nitrogen (TN), ammonia-nitrogen (*NH*_4_^+^−*N*) , nitrate nitrogen (*NO*_3_^−^−*N*) , available phosphorus (AP), total organic carbon and pH value were measured as previously described by Du et al. (Du et al., 2021).

### DNA extraction and high-throughput sequencing of 16S rRNA gene and ITS

A total of 48 samples were sequenced following the procedure below. Total DNA was extracted using the FastDNATM SPIN kit (MP Biomedicals). DNA concentration and quality were assessed by a NanoDrop Spectrophotometer (Nano-100, Aosheng Instrument Co. Ltd). V3-V4 region of 16S rRNA gene was amplified using the primer pair 515F (5’-GTGYCAGCMGCCGCGGTAA-3’)/806R (5’-GGACTACNVGGGTWTCTAAT-3’). The ITS2 region was amplified using the primer pair 5.8F (5’-AACTTTYRRCAAYGGATCWCT-3’)/4R (5’-AGCCTCCGCTTATTGATATGCTTAART-3’). Self-designed 12 bp barcodes were added to the primers to distinguish between samples. A PCR reaction volume of 50 μl was performed with 5μl 10× PCR Buffer (Takara, Dalian, China), 1 μl DNA template (20-30 ng), 1.5 μl of each primer (10 μl mol/L), 1.5 μl dNTPs mixture, 0.5 μl Taq DNA Enzyme (TaKaRa, Beijing, China) and 39 μl ddH_2_O. Amplification for bacterial 16S rRNA gene was performed using the following conditions: 94 C for 1 min; following 30 cycles of 94°C for 20 s, 57°C for 25 s, and 68°C for 45 s, with a final elongation step at 68°C for 10 min, and finally stored at 4°C. Amplification for fungal ITS gene was performed using the following conditions: 94 C for 1 min; following 35 cycles of 94°C for 20 s, 57°C for 25 s, and 68°C for 45 s, with a final elongation step at 68°C for 10 min, and finally stored at 4°C.

The PCR products were detected by electrophoresis on a 1% agarose gel and purified using the E.Z.N.A.TM Gel Extraction Kit (Omega BioTek, Norcross, USA). The amplicons were pooled together in equimolar amounts and the mixed samples were used to prepare the sequencing library with VAHTS™ Nano DNA Library Prep Kit for Illumina according to the MiSeq Reagent Kit Preparation Guide (Illumina). The samples were sequenced using a Miseq platform at Magigene Biotechnology Co., Ltd. (Guangzhou, China). The Illumina sequence reads were deposited in China National Microbiology Data Center (NMDC) with accession numbers (NMDC10018152).

### Sequence preprocessing and bioinformatics approaches

All the sequence preprocessing was conducted by an in house-pipeline (http://mem.rcees.ac.cn:8080) integrated with these bioinformatics tools (Feng et al., 2017). The barcodes were used to assign the raw reads to samples, with one mismatch allowed. Next, both forward and reverse primers and the barcode sequences were trimmed, then pair-ended sequences were merged and the quality checked by Flash program (Magoc & Salzberg, 2011). The Btrim program (Y. Kong, 2011), with threshold of Quality Score > 20 and 5 as window size, was used to filter out unqualified sequences, and sequences with length < 200 bp were also deleted. UPARSE algorithm (Edgar, 2013) was used to remove chimeras and generate OTUs (operational taxonomic units) table at a 97% similarity level without any singletons being discarded.

### Ecological and statistical analysis

Core OTUs in the bacterial and fungal communities were defined using the criteria of Mahoney et al. (Mahoney et al., 2017). Only OTUs present in 95% of all samples were considered to be core OTUs. Based on the resampled OTUs tables of 16S rRNA and ITS gene with 34,619 and 26,693 sequence, respectively, we calculated Chao and phylogenetic diversity index and non-metric multidimensional scaling (NMDS) of potato plant soil communities. Dissimilarity test was calculated to compare within- and between-group similarity through a Jaccard dissimilarity distance matrix by using MRPP (Multi Response Permutation Procedure), ANOSIM (Analysis of Similarities), and PERMANOVA (Permutational multivariate analysis of variance) methods. One‐sample Student’s *t* test was used to measure the significance between the empirical networks and the random networks properties, and the significance of robustness. Mantel test was used to calculate the correlation between microbial communities with soil properties and yield. The differences of diversity and cohesion value among the eight potato cultivar soils were measured by Analysis of Variance (ANOVA) implemented through least significant difference (LSD) test and Tukey *post-hoc* tests in SPSS software. *P* < 0.05 was considered to be significant.

### Intra-domain and inter-domain network analysis and visualization

To elucidate microbial interactions in different cultivar soils, we constructed phylogenetic MENs *via* a Random Matrix Theory (RMT)-based approach in molecular ecological network analysis pipeline (MENA, http://ieg2.ou.edu/MENA) (Deng et al., 2012). Compared with other methods of network construction, the distinguishing feature of this method is that the network is automatically defined and is robust to noise (Deng et al., 2012). To further elucidate the interactions between bacteria and fungi among the soils of the eight potato cultivar, bipartite-networks were calculated *via* the SparCC approach, which can infer correlation with high accuracy from compositional data, based on the IDENAP (http://mem.rcees.ac.cn:8081) workflow (Feng et al., 2019; Feng et al., 2022). The topological roles were defined by two parameters, within-module connectivity (*Zi*) and among module connectivity (*Pi*) and according to those values the roles of nodes were sorted into four subcategories: peripherals, connectors, module hubs, and network hubs (Olesen et al., 2007). In general, module hubs, connectors and network hubs are regarded as the keystone species in molecular ecological networks. The networks were visualized using Gephi 0.9.2 software.

### Network stability and complexity and community assembly

Robustness (Dunne et al., 2002a; Montesinos-Navarro et al., 2017) was calculated. First, the equation:


$$wMIS_{i}=\frac{\sum_{j\neq i}b_{j}s_{ij}}{\sum_{j\neq i}b_{j}}$$

was used to calculate the abundance-weighted mean interaction strength of node *i* where *b_j_* is the relative abundance of species j and *s_ij_* is the association strength between species *i* and *j*, which is measured by the Pearson correlation coefficient, second, by removing nodes with wMIS*_i_* value ≤ 0 from the network, and finally the proportion of remaining nodes was reported as the network robustness.

The cohesion test, which is an abundance-weighted, null model-corrected metric based on pairwise correlations across taxa, is a powerful tool to calculate network complexity (Herren & McMahon, 2017). Positive cohesion, derived from positive pairwise correlations, could reflect the degree of cooperative behaviors in a sample, whereas negative cohesion could indicate the magnitude of competitive behaviors among community members (Yuan et al., 2021). Positive correlations can be the result of facilitation/mutualism among taxa reflecting ecological or functional similarity (Barberán et al., 2012; Durán et al., 2018), and negative correlations can result from competition reflecting with divergent niche requirements among taxa (Zelezniak et al., 2015; Freilich et al., 2018). Two cohesion values (positive and negative) were calculated using the following equations:


$$c_{j}^{pos}=\sum_{i=1}^{n}a_{i}∙{\bar{r}}_{i,r}>0\;\left(Positive\;Cohesion\right)$$

And


$$c_{j}^{neg}=\sum_{i=1}^{n}a_{i}∙{\bar{r}}_{i,r}<0\;\left(Negative\;Cohesion\right)$$

Where *a_i_* is the abundance of OTU *i* in the sample *j* and ${\bar{r}}_{i,r}$ is the connectedness.

To defining the relative importance of community assembly process (*i.e.*, selection, dispersal, diversification and drift), a new mathematical framework named iCAMP (the infer community assembly mechanism by phylogenetic-bin-based null model analysis) was used (http://ieg3.rccc.ou.edu:8080) (Ning et al., 2020). The specific rationale of iCAMP is that in order to quantify various ecological processes, the observed taxa were first divided into different groups (*i.e.*, ‘bins’) based on their phylogenetic relationships. Then, the process governing each bin is identified based on null model analysis of the phylogenetic diversity using beta Net Relatedness Index (βNRI), and taxonomic β-diversities using modified Raup–Crick metric (RC).

## Results

### Soil properties and yields of different potato cultivars under continuous cropping regime

ANOVA analysis indicated that there were significant differences (*P* < 0.05) of soil properties and yield among the eight potato cultivars (**Table 1**). The highest TP, TN and AP concentration were observed in cultivars ZS5 and HS4, while the highest *NH*_4_−*N* concentration was observed in cultivar XJ2, the highest *NO*_3_−*N* concentration was observed in cultivar HC1, and the highest TOC content was observed in cultivar HS4. The pH value did not exhibit significant differences among the eight cultivar soils. On the basis of yi eld, cultivar ZS5 possessed the highest yield out of all of the cultivars under continuous cropping regime.

**Table 1:** Soil properties and potato yield.

Potatocultivar	TP (mg/kg)	TN (mg/kg)	NH_4_-N (mg/kg)	NO_3_-N (mg/kg)	AP (mg/kg)	TOC (%)	pH	Yield (kg/ha)
DN303	3166.50cd	2321.75de	73.67b	233.80b	416.94bc	2.68de	3.80a	19766.40bc
E14	2761.40d	2047.47e	67.52b	241.60b	368.80c	2.20e	3.99a	23457.42b
HC1	3729.71bc	3131.74bc	59.38b	445.09a	412.22bc	4.27ab	3.51a	24187.32b
HE1	3760.68bc	2691.66cd	45.35b	277.78b	426.62abc	3.21cd	3.97a	20517.66bc
HS4	4158.99ab	3551.22ab	108.71ab	402.41ab	444.01ab	4.62a	3.58a	18517.50c
XJ2	3386.90cd	2839.58dc	155.95a	323.06ab	395.65bc	2.34e	3.74a	21806.25bc
ZS5	4337.08a	3701.96a	56.25b	320.77b	486.26a	3.86bc	3.95a	30156.22a
ZS4	3312.45bc	2316.50de	79.9ab	295.6ab	457.53ab	2.61de	3.99a	15905.70d

Dongnong303 (DN303), E14(E14), Huacai1 (HC1), Huaen1 (HE1), Huashu4 (HS4), Xingjia2 (XJ2), Zhongshu5 (ZS5) and Zhongshu4 (ZS4); soil total organic matter (OM), and the contents of total nitrogen (TN), ammonia-nitrogen (NH_4_+-N), nitrate nitrogen (NO_3_−-N), total phosphorus (TP), and available phosphorus (AP). Different letters in the table represent significant differences (*P < 0.05*). Same as below.

### Soil bacterial and fungal community diversity, structure and composition of different potato cultivar soils under continuous cropping regime

Using a similarity threshold of 97%, there were 14, 740 OTUs for bacteria (2, 375, 667 sequences) and 1, 531 OTUs for fungi (1, 892, 836 sequences). Chao and phylogenetic diversity indices of bacterial and fungal communities were calculated to discover the variation of alpha diversity among the eight potato cultivar soils under continuous-cropping regime. The results showed significant differences among the soil bacterial and fungal communities of the eight cultivar soils (*P* < 0.05) (**Figure S2**). Non-metric multidimensional scaling (NMDS) based on Jaccard distance of 16S rRNA (stress = 0.168) and ITS (stress = 0.114) genes (**Figure S3**), and dissimilarity analysis results (**Tables S1**, **S2**) all showed that there were significant differences (*P* < 0.05) among the soil community structures of the eight potato cultivars. Overall, the potato cultivars reshaped the bacterial and fungal communities’ structure and diversity under continuous-cropping regime.

For the bacterial community, all of the sequences obtained from the 48 samples could be classified into 32 phyla and 785 genera. The dominant bacterial phyla (relative sequence abundance > 1%) across all potato soil samples were *Proteobacteria* (58.1%), *Acidobacteria* (14.6%), *Actinobacteria* (9.9%), *Bacteroidetes* (5.8%), *Firmicutes* (3.1%), *Planctomycetes* (1.7%) and *WPS-2* (1.2%) accounting for 94.3% of the bacterial sequences (**Figure S4A**), while the dominant bacterial genera (relative sequence abundance > 1%) across all potato soil samples were *Acidobacterium*, *Rhodanobacter*, *Burkholderia*, *Acidisoma*, *Mycobacterium*, *Conexibacter*, *Arachidicoccus*, *WPS-2*, *Vampirovibrio*, *Alkanibacter*, *Rhizomicrobium*, *Thermogutta*, *Gp14*, *Gp13*, *Gp3*, *Subdivision3 genera incertae sedis*, *Achromobacter*, *Yersinia* and *Paenibacillus* (**Figure S4B**). The dominant fungal phyla were *Basidiomycota* (52.6%) and *Ascomycota* (47.0%), accounting for 99.4% of the fungal sequences (**Figure S4C**), while the dominate fungal genera (relative sequence abundance > 1%) across all potato soil samples were *Eupenicillium*, *Hypocrea*, *Trechispora*, *Humicola*, *Nectria*, *Neurospora*, *Talaromyces*, *Cyphellophora*, *Gibberella*, *Emericellopsis*, *Simplicillium*, *Thielavia*, *Schizoblastosporion* and *Sphaerobolus* (**Figure S4D**).

### Intra-domain networks analysis for soil microbial communities of different potato cultivars under continuous cropping regime

**Figure 1:**
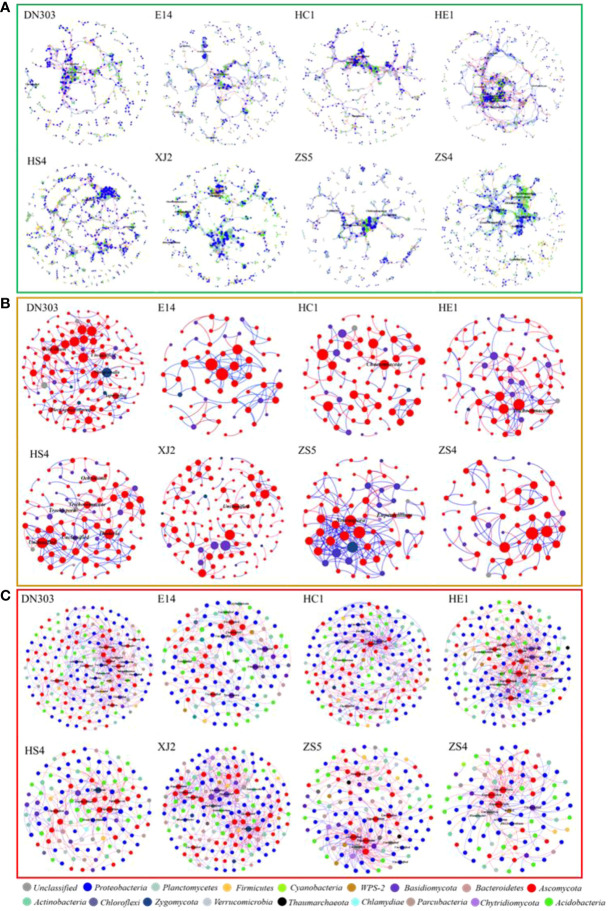
Intra- and inter- domain networks of bacterial and fungal and bacterial-fungal communities. The size of the node represents the node degree of the OTUs (The number of connections among OTUs). Node color represents taxonomic groups within each network. Lines connecting nodes (edges) represented positive (red) and negative (green) interactions. Those nodes with a label at genus level were core taxa of the network (connectors, module hubs and network hubs). **(A)** The intra-domain networks of the bacterial community. **(B)** The intra-domain networks of fungal community. **(C)** The inter-domain networks of bacterial-fungal community. Dongnong303 (DN303), E14(E14), Huacai1 (HC1), Huaen1 (HE1), Huashu4 (HS4), Xingjia2 (XJ2), Zhongshu5 (ZS5) and Zhongshu4 (ZS4) were planted under continuous cropping regime. The networks were visualized using Gephi 0.9.2 software.

Molecular ecological networks (MENs) were constructed to explored the changes of microbial inter-relationship in soil microbial communities of different potato cultivars under continuous-cropping regime (**Figures 1A, B**). MENs of all cultivars were constructed with a similar threshold, so that the topology coefficients of different networks could be directly compared. The overall topology indices revealed that all curves of network connectivity distribution fitted well with the power-law model (R^2^ values of bacterial community were from 0.699 to 0.808, R^2^ values of fungal community were from 0.603 to 0.823) (**Tables S3**, **S4**), indicating the MENs were scale-free (Barabási, 2009). The average path lengths (GD) were from 7.308 to 13.564 for the bacterial networks and from 2.381 to 5.85 for the fungal empirical networks, which were close to logarithms of the total number of network nodes and higher than those of their corresponding random networks, suggesting that the MENs in these microbial communities had the typical property of small world (Duncan & Steven, 1998), and the modularity for soil bacterial and fungal networks of different potato cultivars ranged from 0.735 to 0.896 and from 0.461 to 0.851, respectively, which was significantly higher than the modularity value of their corresponding randomized networks, indicating that all of the constructed networks possessed modular topology. The networks of each cultivar soil were divided into 45-81 and 7-20 (**Table S3**) discrete modules containing closely related microbial taxa for bacterial networks and fungal networks, respectively. All these key topological properties qualified the constructed networks for further analysis.

**Figure 2:**
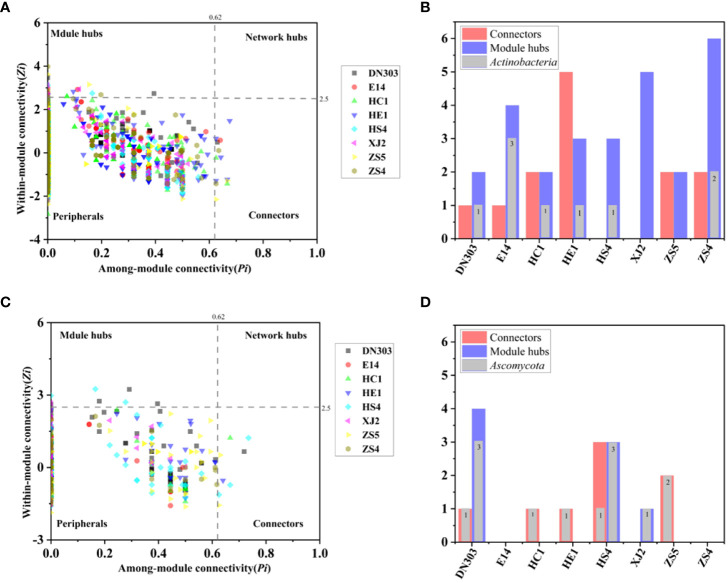
Zi-Pi plot and core node distribution of each cultivar. **(A, B)** Zi-Pi plot of bacterial community. **(C, D)** Zi-Pi plot of fungal community. The digits presented in panels **(B, D)** represent the number of *Actinobacteria* and *Ascomycota* in the core node. All images were generated by Origin 2021 software.

Node plays different topological roles within a network, and the analysis of these roles within modules is of great importance for identifying keystone microbial species. In order to identify the topological roles of key nodes in the network, we divided the nodes into four subcategories types according to their intra-module and inter-module connectivity values: peripherals, connectors, module hubs, and network hubs. For the bacterial community, we found different connectors or module hubs (3-8 nodes) in each network. Module hubs were highly connected to many other nodes within their respective modules, and could be considered as keystone nodes in the entire network. Module hubs of individual networks from different potato cultivars were observed: DN303 possessed 2 module hubs, E14 possessed 4, HC1 possessed 2, HE1 possessed 3, HS4 possessed 3, XJ2 possessed 5, ZS5 possessed 2, and ZS4 possessed 6 (**Figures 2A, B**; **Table S5**). Of these module hubs, 9 belonged to *Actinobacteria*, 8 to *Proteobacteria*, 3 to *Firmicutes*, 2 to *Acidobacteria*, 2 to *Bacteroidetes*, 1 to *WPS-1*, 1 to *Verrucomicrobia* and 1 node belonged to *Unclassified*. In this study, 1 node, found as a connector or module hub, belonging to the phylum *Actinobacteria* was observed in almost all cultivar soil networks (cultivars DN303, E14, HC1, HE1, HS4 and ZS4). Therefore, the phylum *Actinobacteria* might be the most important keystone taxon in potato soils under continuous cropping regime. For the fungal community, we found that different connectors or module hubs (1-6 nodes) presented in different networks, except for networks of cultivars E14 and ZS4. We also observed module hubs in several networks: DN303 possessed 4, HS4 possessed 3, and XJ2 possessed 1 (**Figures 2C, D**; **Table S6**). Of these module hubs, 7 belonged to *Ascomycota* and 1 belonged to *Basidiomycota*. In this study, 14 connectors and module hubs were observed across all networks and belonged to phylum *Ascomycota*, and were observed in almost all cultivar networks (cultivars DN303, HC1, HE1, HS4, XJ2 and ZS5). Thus, the phylum *Ascomycota* may be the most important keystone fungal group in potato soils under continuous cropping regime.

### Inter-domain networks analysis between bacterial and fungal communities of different potato cultivars under continuous cropping regime

To analyze the inter-domain interaction between bacterial and fungal communities of the eight potato cultivar soils, the Inter-Domain Ecological Networks (IDENs) approach (Feng et al., 2019) was implemented. Through the IDENs construction process, a total of 79 to 121 bacterial and 30 to 52 fungal OTUs were eventually chosen to illustrate the bacteria-fungi associations in the eight potato cultivar soils with 169 to 502 observed links in the networks (**Table S7**). These bacterial-fungal inter-domain networks showed some basic network topological features, such as nestedness and modularity. The connectance for these IDENs was 0.059 to 0.100, showing that 5.9% to 10% of possible links were observed as bacteria-fungi associations. The web asymmetry of these networks was -0.362 to -0.959, indicating a skewed richness pattern for bacterial and fungal nodes in these networks. Even so, there were still 1.520 to 2.902 links connected per OTUs on average. Together the above topological property information demonstrated that these eight bipartite networks were obviously different. All of the bipartite networks were primarily composed of *Proteobacteria*, *Acidobacteria* and *Ascomycota* nodes (**Figure 1C**; **Table S8**). To further explore the interactions between bacteria and fungi, the keystone microorganisms were also extracted. The results showed that most connector hubs belonged to bacteria and most module hubs were fungi, indicating that bacteria were the dominant linker of the two communities.

### Networks stability and complexity of bacterial and fungal communities of different potato cultivars under continuous cropping regime

**Figure 3:**
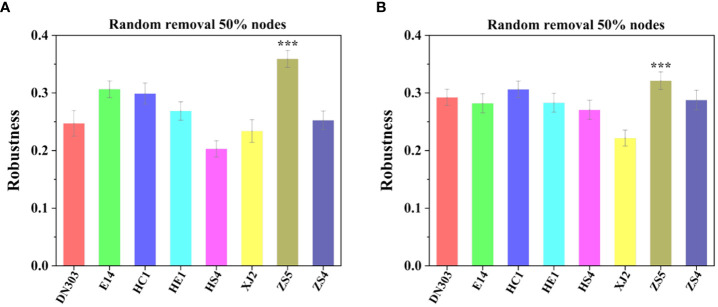
Robustness analysis for bacterial and fungal communities among the eight potato cultivars under continues cropping regime. Robustness measured as the proportion of taxa remaining after 50% of the taxa are randomly removed from each of the empirical MENs. **(A)** The robustness of bacterial communities. **(B)** The robustness of fungal communities. All model and images were calculated and generated by RStudio software. "***" mean the significantly differed at the level of P< 0.001.

**Figure 4:**
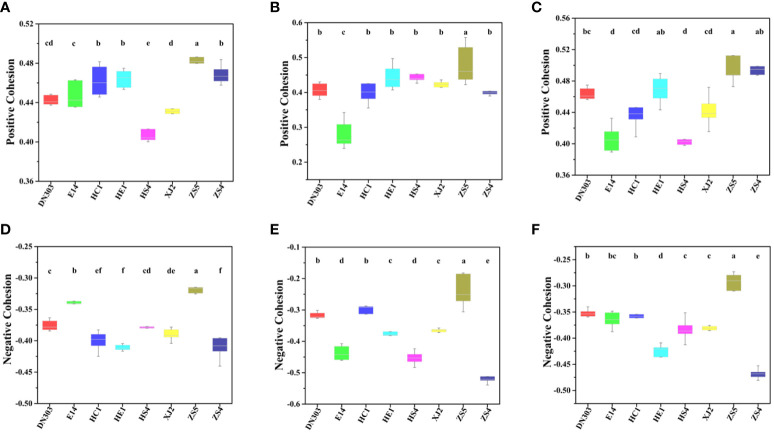
Difference in cohesion value of bacterial, fungal and bacterial-fungal communities among the eight potato cultivars. The difference was calculated by ANOVA analysis, the same letter indicates no difference (*P* < 0.05). **(A)** The bacterial community positive cohesion, **(B)** the fungal community positive cohesion, **(C)** the bacteria-fungi community positive cohesion, **(D)** the bacterial negative cohesion, **(E)** the fungal negative cohesion, and **(F)** the bacteria-fungi community negative cohesion.

Topological stability of the community was calculated from the empirical data to elaborate the effect of continuous cropping regime on the soil microbiomes of different potato cultivars. Robustness of a MEN is defined as the proportion of species remaining in the network after random or target node removal (Deng et al., 2012; Yuan et al., 2021). For random species removal simulations, 50% of the nodes were randomly removed. The results showed that cultivar ZS5 possessed highest robustness value for both the bacterial (0.3591 ± 0.0147) and fungal communities (0.3213 ± 0.0523), indicating the highest topological stability among the eight potato cultivar soils under continuous cropping regime (**Figure 3**). To further evaluate the degree of cooperation/competition between microorganisms of bacterial, fungal and bacterial-fungal communities, a new index named cohesion was implemented (**Figure 4**). The results showed that the positive cohesion value of cultivar ZS5 was the highest, the highest negative cohesion value was also found in cultivar ZS5, meaning this soil community possessed the highest cooperation and a lowest competition. In summary, these results might indicate that cultivar ZS5 possessed the highest resistance to continue cropping obstacle among eight potato cultivars.

### Assembly process of bacterial and fungal communities of different potato cultivars under continuous cropping regime

**Figure 5:**
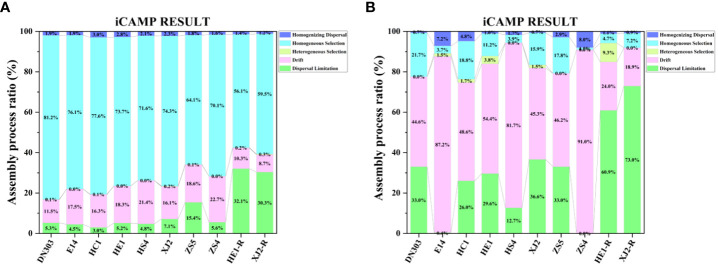
Ecological processes of microbial community assembly. **(A)** The bacterial community, **(B)** the fungal community. Dongnong303 (DN303), E14(E14), Huacai1 (HC1), Huaen1 (HE1), Huashu4 (HS4), Xingjia2 (XJ2), Zhongshu5 (ZS5) and Zhongshu4 (ZS4) were planted under continuous cropping regime. Huaen1 (HE1-R) and Xingjia2 (XJ2-R) were planted under rice-potato rotation regime. Different colors represent the different assembly processes.

To gain a better understanding of community assembly, we used iCAMP to infer community assembly processes from phylogenetic data. Our results exhibited that homogeneous selection was the most important ecological process driving bacterial community assembly for the eight cultivars under continuous cropping regime and the two cultivars under rotation regime (**Figure 5A**). However, the percentage of homogeneous selection under continuous cropping (64.1% - 81.2%) was significantly higher than under rotation regime (56.1% - 59.5%), while the cultivar ZS5 (64.1%) possessed lowest percentage of homogeneous selection among the eight cultivar soils (70.1% - 81.2%) under continuous cropping regime. Drift was the most important ecological process driving fungal community assembly in the eight cultivars under continuous cropping regime (44.6% - 91.0%), and dispersal limitation (60.9% - 72.9%) was the most important ecological process driving fungal community assembly in the two cultivars under rotation regime (**Figure 5B**). These results indicated that continuous cropping regime mainly influenced the bacterial community assembly process.

## Discussion

Continuous cropping obstacle is a major factor that decreases crop yield and quality (Qin et al., 2017), and choosing a suitable cultivar which possesses a higher resistance to continuous cropping obstacle is an effective and feasible way to maximize yield and quality (Lu et al., 2013). By studying the stability and complexity, as well as the assembly process, of soil microbial communities from different cultivars, it could help us better understand the role that the microbiome plays in the process of resistance to continuous cropping obstacles, and provide a theoretical and experimental basis for alleviating continuous cropping obstacles. In this study, we surveyed eight continuous cropping and two rotation regime experimental plots to study the bacterial and fungal communities’ stability and complexity, and assembly process’ response to potato cultivar and continuous cropping treatments. Network analysis and iCAMP tools helped us to gain an integrated understanding of the microbial community assembly rules reflecting ecological processes in the continuous-cropping potato soil system, such as abiotic selection (*i.e.*, environmental filtering) and biotic selection (*i.e.*, cooperation, competition, and niche partitioning) (Layeghifard et al., 2017), dispersal and drift (Ning et al., 2020).

### Continuous cropping regime influence potato soil microbial communities’ structure and composition by environmental filter and biotic selection

Previous studies indicated that not only the plant species but also the cultivar influenced the soil microbial community structure and composition (Hardoim et al., 2011; Huang et al., 2017; Jiang et al., 2017), our study found a similar phenomenon, in that there were significant difference among soil bacterial and fungal communities of the eight potato cultivars under continuous cropping regime (**Figures S2-S4**). The reasons for the different diversity, structure and composition of the bacterial and fungal communities may include the results of the selective assembly process in soil of different plant cultivars (Mendes et al., 2014). The organizing principle for the establishment of unique microbiota that possess probiotic functions for plant growth and health under abiotic and biotic conditions is likely explained by two mutually non-exclusive mechanisms (Mendes et al., 2014). Firstly, the free-living microbial species spontaneously responded to root derived signals of different plant cultivars. Some researchers have discovered that plant cultivars assemble their own rhizosphere soil microbial community through the selection of microorganism by different root exudates (İnceoğlu et al., 2010; Micallef et al., 2009). Our results showed that bacterial and fungal communities of the eight potato cultivars possessed significantly different assembly processes (**Figure 5**). Second possibility is that the interactions between microorganisms provided an advantage for dominant microbiota co-colonization and selected for host-adapted microorganisms that impact plant fitness. Moreover, the rhizosphere soils containing prokaryotic and eukaryotic microbes have evolved and filled with a myriad of cooperative and competitive interaction mechanisms that shape and potentially stabilize microbial assemblages (Hassani et al., 2018), these may be the dominant reasons that shaped the differences in intra- and inter- domain networks structure for the different potato cultivar soils (**Figure 1**). Therefore, selective pressure from both abiotic and biotic factors acting on holobiont components has likely shaped plant-associated microbial communities (Hassani et al., 2018).

### Continuous cropping mainly caused change among rare taxa while core taxa were more likely dominated by the biotic selection

For bacterial communities, *Proteobacteria*, *Acidobacteria*, *Actinobacteria*, and *Bacteroidetes* were essential members across all cultivars (**Figure S4**), which is in agreement with previous studies of potato plants under continuous-cropping regime (Mardanova et al., 2019; Neilson et al., 2020; Qian et al., 2020). The most dominant phylum in bacterial community was *Proteobacteria*, with an average abundance of more than 58.1% in continuous cropping potato samples. For the fungal communities, *Ascomycota* and *Basidiomycota* were the most dominant fungal phyla, accounting for more than 95% of fungi across all cultivars (**Figure 1**). The bacterial and fungal, and bacterial-fungal networks, were also mainly dominated by *Proteobacteria*, *Acidobacteria*, *Actinobacteria*, *Bacteroidetes*, *Ascomycota* and *Basidiomycota*, which accounted for more than 90% of the nodes across all networks. Generalists assigned to different module hubs and connectors were considered to be the keystone nodes in networks (Zhou et al., 2011; Jiang et al., 2015). The keystone node would be an essential determinant for the key functions colonization of microbial taxa in soil (Jiang et al., 2017). In our study, the phyla *Proteobacteria* and *Actinobacteria* were the keystone taxa for all of the continuous-cropping potato soil bacterial communities (**Figures 1**, **2**, **Table S5**), The phylum *Proteobacteria* is always abundant in plant soil because of their generally fast-growing *r*-strategist lifestyle and ability to utilize a wide range of root-derived carbon substrates (Fierer et al., 2007; Lauber et al., 2009; Peiffer et al., 2013), while *Actinobacteria* are the main source of nutrients in soil and may possess the potential to produce antibacterial and nematocidal compounds to prevent some soil-borne diseases, such as soybean root rot and potato scab (Franche et al., 2008; Shi et al., 2019). All fungal networks showed that the phylum *Ascomycota* was the dominant taxon for continuous cropping potato soil communities (**Figures 1**, **2**, **Table S6**), which is a large, diverse group of fungi that are capable of a wide variety of metabolic pathways that function directly in maintaining soil ecosystem function and nutrient cycling (Manici & Caputo, 2009; Bulgarelli et al., 2012; Shen et al., 2018; Zimudzi et al., 2018). Overall, although continuous cropping can lead to differences in diversity (Lu et al., 2013; Qin et al., 2017), the core microbiome of different cultivars remained essentially the same in our study (**Figure S4**), suggesting that continuous cropping might mainly cause changes among the rare taxa of the microbial communities (Qian et al., 2020). Moreover, network analysis mainly explained the biotic selection part of the selection process (Faust, 2021), and the results also revealed that the taxa rarely exist in empirical networks (**Figure 1**). This result indicated that, during the process of community assembly, the rare taxa might be dominated by environmental filtering, and that environmental stress was mainly influenced by the cultivars’ exudates under continuous cropping (Micallef et al., 2009; İnceoğlu et al., 2010), and the core taxa were more likely dominated by the biotic selection (bacteria-bacteria, fungi-fungi and bacteria-fungi correlations) after the environmental filtering.

### The higher homogeneous selection process of bacterial community may trigger continuous cropping obstacles

Homogeneous selection means that the selection under homogeneous abiotic and biotic environmental conditions will lead to more similar structures among communities (J. Zhou & Ning, 2017) Our result showed that the bacterial community was mainly dominated by deterministic processes (*i.e.*, homogeneous selection) (**Figure 5A**), while the fungal community was mainly dominated by stochastic processes (*i.e.*, drift and dispersal limitation) (**Figure 5B**). A previous study showed that deterministic processes display relatively stronger effects on the assembly of bacterial communities, while bacteria possess wider niche breadth, higher abundance, more widespread, a smaller body size and a higher dispersal rate as compared to fungal communities (Powell et al., 2015). The smaller body size has the ability of a higher dispersal rates and faster population growth rates, which can lead to relatively stronger deterministic processes through better ability to disperse to new habitats and faster establishment. (Aslani et al., 2022). In contrast, a lower dispersal rate may hamper the ability of species to colonize various environmental conditions, thereby reduce the impact of environmental selection on community assembly. (Leibold et al., 2004). Thus, to offset the greater probability of dying out (Fodelianakis et al., 2021), fungi show more stochastic distribution patterns (Nemergut et al., 2013; Zinger et al., 2019). These results suggesting that the continuous copping regime mainly influenced the bacterial communities (*i.e.*, homogeneous selection process), which was consistent with the Mantel test results (**Table 2**). Interestingly, by comparing continuous cropping and rotation regimes, the iCAMP results showed that the deterministic process of the bacterial community under continuous cropping regime was higher than under rotation regime, and was dominated by homogenous selection. Therefore, we infer that the higher homogeneous selection process of the bacterial community might be the cause of continuous cropping obstacles. As cultivar ZS5 exhibited the lowest percentage of homogeneous selection among the cultivar soils under continuous cropping regime, it may suggest a high resistance to continuous cropping obstacles.

**Table 2:** Mantel test based on Jaccard distance between bacterial and fungal communities with soil properties and potato yield.

Soil community	Jaccard-distance	TP(mg/kg)	TN(mg/kg)	NH4-N (mg/kg)	NO_3_-N(mg/kg)	AP(mg/kg)	TOC(%)	pH	Yield
Bacteria	r	0.0767	0.1559	0.176	0.0743	0.0007	0.1929	-0.0076	0.2726
	*P*	0.128	0.009**	0.041	0.227	0.491	0.009**	0.518	0.001***
Fungi	r	-0.0039	0.0414	0.1114	-0.0542	0.0076	0.0041	0.0149	-0.042
	*P*	0.502	0.214	0.124	0.757	0.45	0.464	0.4	0.746

The significantly correlated at the level of 0.0001***, 0.01**, and 0.05*.

### Microbial communities’ high stability and complexity provide a high resistance to continuous cropping obstacle

There are still some fundamental, yet debated, questions surround whether and how the stability of an ecosystems depend on its complexity (Montoya et al., 2006; Hillebrand et al., 2018; Solé & Montoya, 2001; Okuyama & Holland, 2008). A systems-analysis approach is often essential for acquiring an understanding of all the dynamical feedbacks at the ecosystem level (Landi et al., 2018), while a constructed network can be used to simplify the vast complexity of a real community, to formally and effectively describe and investigate ecological phenomena, and to understand how ecosystems react to stress and perturbations (Dunne et al., 2002a). Here we calculated communities’ stability and complexity by robustness and cohesion value. We conducted a robustness test which was defined as the proportion of remaining species in this network after node removal (Dunne et al., 2002a; Montesinos-Navarro et al., 2017) to measure the resistance of a network through natural connectivity changes under node or edge attacking (Albert et al., 2000; Peng & Wu, 2016), and calculated a different metric named cohesion, which is an abundance-weighted, null model-corrected metric based on pairwise correlations across taxa (Herren & McMahon, 2017; Yuan et al., 2021).

In this study, the higher stability of potato soil microbial communities was likely due to their higher complexity under continuous cropping. The results showed that cultivar ZS5 possessed the highest robustness of bacterial and fungal communities, while the highest positive cohesion value and highest negative cohesion were also observed in cultivar ZS5 for all groups (bacterial, fungal bacterial-fungal communities), indicating that the complexity exhibited positive contribution to communities’ stability under continuous cropping regime. In the community, positive associations of bacteria-bacteria, fungi-fungi and bacteria-fungi may have beneficial effects on microorganism growth, resistance or colonization and micro-ecosystem stability, while either some bacteria or fungi would be inhibited or harmed by negative associations (Feng et al., 2019). The higher degree of positive associations means the stronger cooperative and trophic interactions between functional groups of soil microflora such as commensalism, mutualistic interactions, syntrophic interactions and cross-feeding as well as shared environmental requirements and common dispersal barriers (Yuan et al., 2021), which was related with the increase in resource availability (Morriën et al., 2017; Banerjee et al., 2019). By contrast, negative associations could reflect mainly competition for limiting resources as well as distinctive environmental niches and spatial isolation (Berry and Widder, 2014; Fuhrman, 2009). As revealed by our Mantel test (**Table 2**), the bacterial community was significantly (*P* < 0.05) associate with TN and TOC, while the cultivar ZS5 possessed the highest TN concentration, and the third highest TOC concentration among all of the potato cultivar soils, but there was no significant correlation of the fungal communities with any environmental factors. That may be cause the smaller microorganisms (i.e., bacteria) to respond more rapidly to environmental change (Korhonen et al., 2010; Vellend et al., 2014). We infer that different potato cultivar soils assemble their microbial communities by root exudates under continuous cropping regime (i.e., environmental filtering) (Hardoim et al., 2011; Qin et al., 2017; Qian et al., 2020), and then the colonized species promote the concentration of soil nutrients through positive interactions (i.e., biotic selection) (positive associations of bacteria-bacteria, fungi-fungi and bacteria-fungi) (Yuan et al., 2021), resulting in a higher ability of bacterial and fungal community to resist environmental change (Hernandez et al., 2021; Wu et al., 2021). These results may explain the reason why cultivar ZS5 possessed the highest stability of all potato cultivars.

Overall, our study demonstrated that the continuous cropping regime mainly influenced the dominant bacterial community’ assembly process by increasing the homogeneous selection process, which may be the main reason for the potato continuous cropping obstacles. Cultivar ZS5 also possessed the highest resistance to continuous cropping obstacle due to its high community stability and complexity, and lower bacterial community homogeneous selection process.

## Conclusion

In the process of bacterial community assembly, continuous cropping mainly influenced the homogeneous selection process, while the assembly of fungal communities exhibited significant influence by stochastic processes under continuous cropping. Moreover, the higher homogeneous selection process of bacterial communities may suggest a more serious continuous cropping obstacle under continue cropping regime. Among the eight potato cultivars that are widely planted across China, cultivar ZS5 exhibited a relatively lower proportion of homogeneous selection process, and a higher stability and complexity, resulting in a higher yield than other cultivars under continuous cropping regime. These results may indicate a higher resistance of cultivar ZS5 to continuous-cropping obstacle than others cultivars. Further studies should strive for a deeper understanding to slow down the deterministic process of community assembly (e.g., by biotic and abiotic selection), and revealed the relationship among key microbial organisms by the network analysis, and their potential functions, and explore the possibility of guiding the potato soil community, which may contribute to enhanced potato production.

## Data availability statement

The data presented in the study are deposited in the China National Microbiology Data Center (NMDC) repository, accession number NMDC10018152.

## Author contributions

QH, LT, XX and YD designed the experiments. SG, QH, XY and XD took samples and performed all data measurement. SG contributed to the data analysis. SG and QH wrote the paper. All authors contributed to the article and approved the submitted version.

## Funding

This research was supported by the key research and development program of Hunan Province, China (2022NK2051), the project of key laboratory of crop germplasm innovation and utilization, Hunan Agricultural University, China (18KFXM02), and Wenshan Tobacco Company of Yunnan Province(2021530000241033) of China.

## Acknowledgments

We thank Dr. James Walter Voordeckers for carefully editing the grammar of the manuscript and for some valuable suggestions for this manuscript.

## Conflict of interest

The authors declare that the research was conducted in the absence of any commercial or financial relationships that could be construed as a potential conflict of interest.

## Publisher’s note

All claims expressed in this article are solely those of the authors and do not necessarily represent those of their affiliated organizations, or those of the publisher, the editors and the reviewers. Any product that may be evaluated in this article, or claim that may be made by its manufacturer, is not guaranteed or endorsed by the publisher.
