# Supplementary material for: Soil microbial community assembly and stability are associated with potato (Solanum tuberosum L.) fitness under continuous cropping regime
Source: Front Plant Sci. 2022 Oct 3;13:1000045. doi: 10.3389/fpls.2022.1000045 (PMC9574259; doi:10.3389/fpls.2022.1000045)
Supplement: Supplementary file 1 [file DataSheet_1.docx]

**Supplementary Material**

# Soil microbial community assembly and stability are associated with potato (*Solanum tuberosum* L.) fitness under continuous cropping regime

Running title: The divergences of bacterial and fungal community in continuous-cropping soils of different potato cultivars

**Songsong Gu ^1, 3^, Xingyao Xiong ^1, 2^, Lin Tan ^1^, Ye Deng ^3^, Xiongfeng Du ^3^, Xingxing Yang ^4^, Qiulong Hu ^1^***

*^1^ Hunan Agricultural University, Changsha, Hunan, China.*

*^2^Agricultural Genomics Institute at Shenzhen, Chinese Academy of Agricultural Sciences, Shenzhen, China*

*^3^Key Laboratory for Environmental Biotechnology, Research Center for Eco-Environmental Sciences, Chinese Academy of Sciences (CAS), Beijing, China.*

*^4^ Hunan Center of Crop Germplasm Resources and breeding crop, Changsha, Hunan, China.*

***Correspondence:**

Qiulong Hu

*Hunan Agricultural University, Changsha, Hunan, China.*

E-mail: [huqiulongnet@126.com](mailto:huqiulongnet@126.com)

**Detailed information of the Supplementary Material**

The number of Figures: 4

The number of Tables: 8

1. **Figures**

Figure S1 Map of sampling site plots.

Figure S2 Diversity indices of the microbial communities. a, c) Bacterial community chao and PD indices. b, d) Fungal community chao and PD indices. Different colors represent different potato cultivars. The same letter indicates no difference (*P > 0.05*).

Figure S3 Nonmetric multidimensional scaling (NMDS) analysis for Jaccard distance. a) Bacterial community, b) fungal community. Different color and shape represent different potato cultivars.

Figure S4 Distribution of microbiome community at phylum and genus level with the relative abundance higher than 1%. a, b) Bacterial community. c, d) Fungal community.

Figure. S1


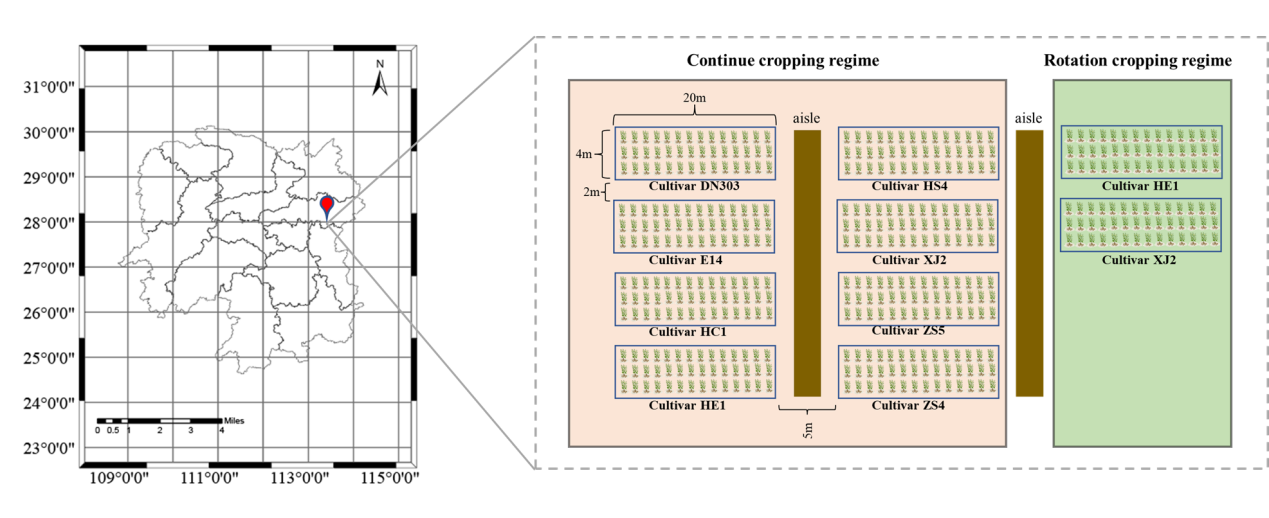


Figure. S2


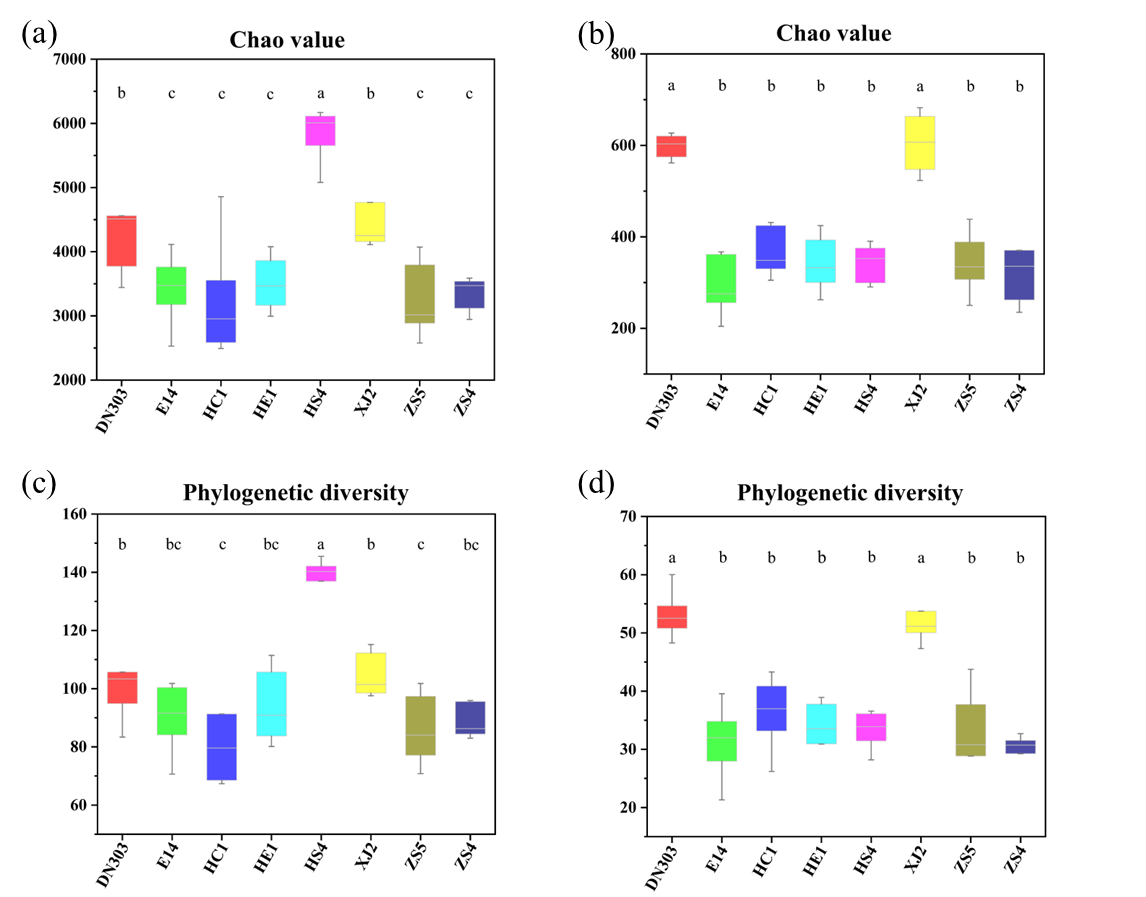


Figure. S3
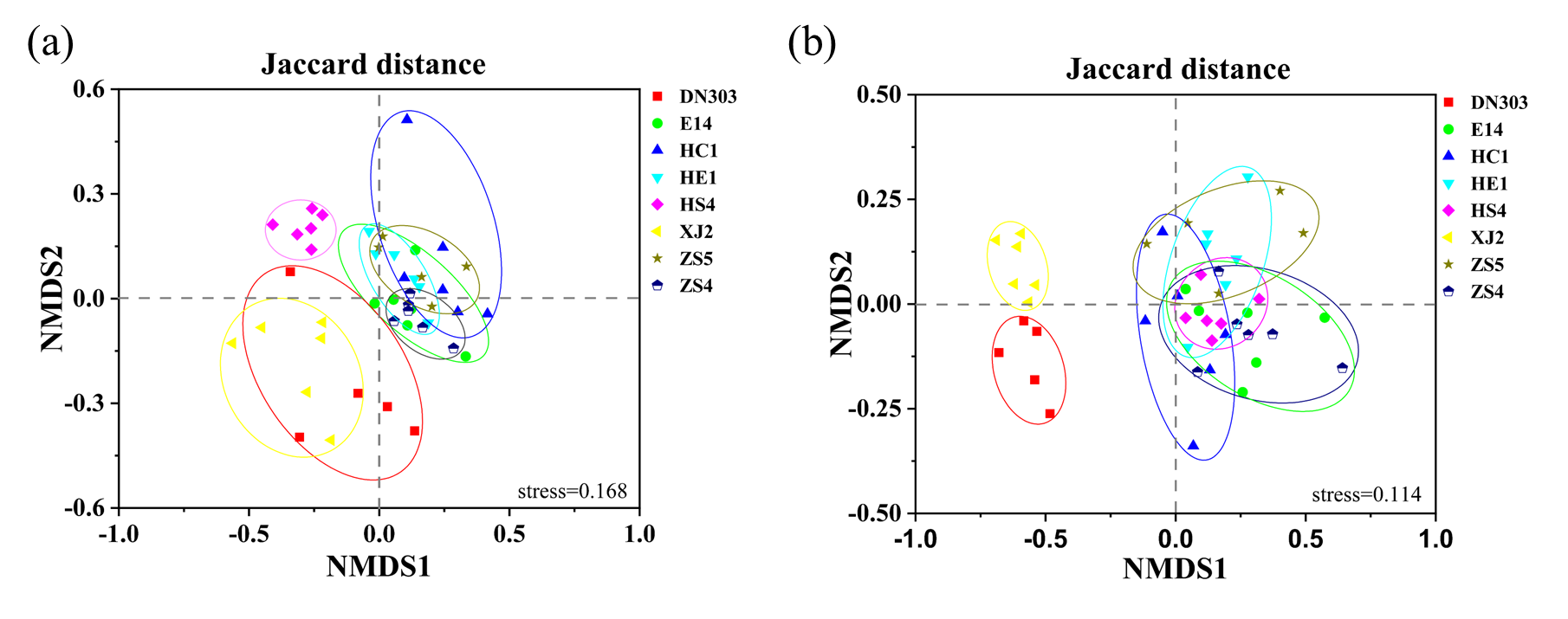


Figure. S4
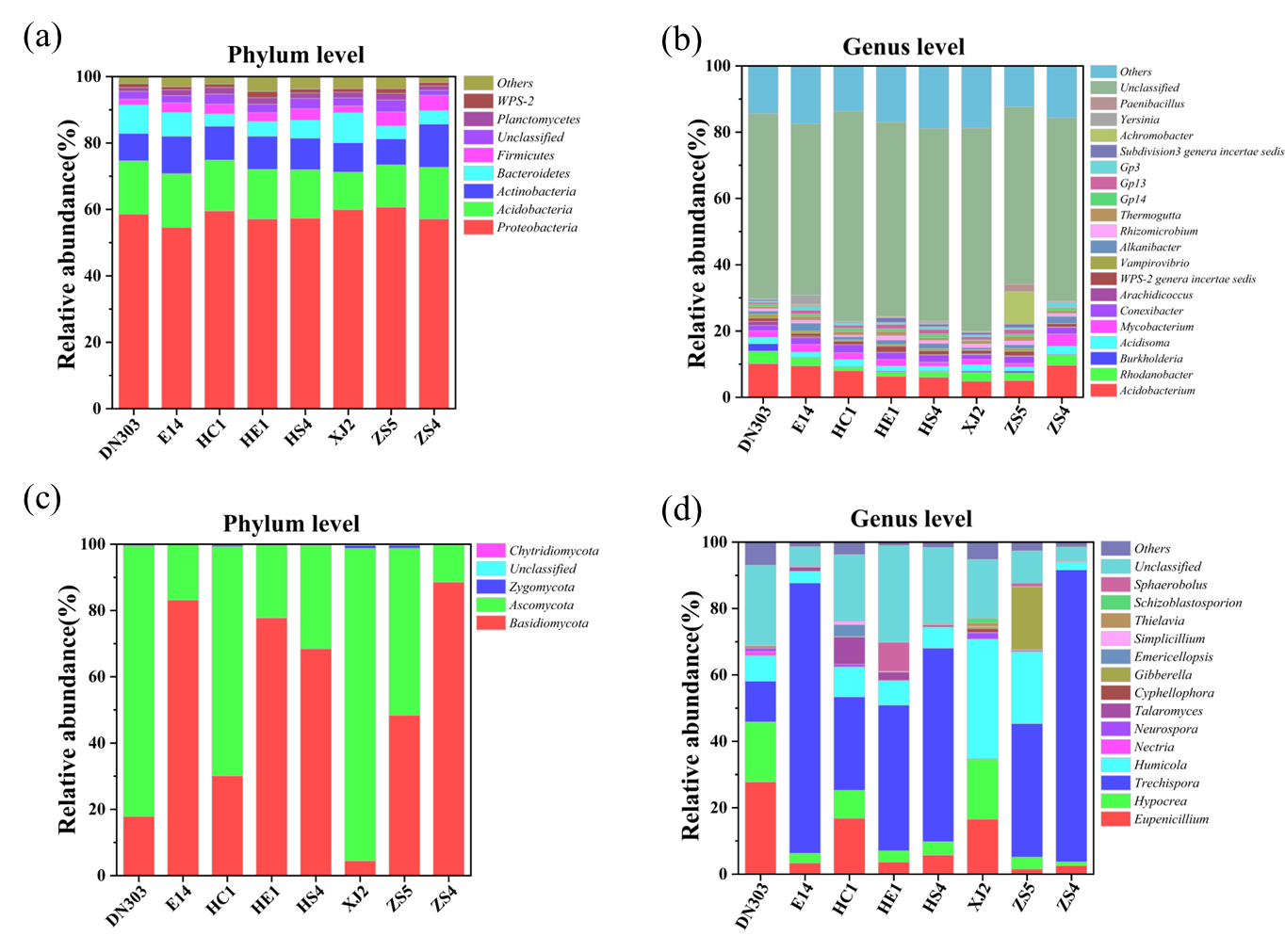


1. **Table**

Table S1 The dissimilarity test of bacterial community structure based on Bray-Curtis distance.

| Dissimilarity result of Bray-Curtis distance | MRPP | | ANOSIM | | PERMANOVA | |
| --- | --- | --- | --- | --- | --- | --- |
|  | Delta | *P* | R | *P* | R^2^ | *P* |
| DN303 VS. E14 | 0.449 | 0.11 | 0.184 | 0.107 | 1.619 | 0.127 |
| DN303 VS. HC1 | 0.433 | 0.041* | 0.219 | 0.065 | 2.307 | 0.023* |
| DN303 VS. HE1 | 0.416 | 0.012* | 0.448 | 0.019* | 3.556 | 0.019* |
| DN303 VS. HS4 | 0.388 | 0.004** | 0.6 | 0.011* | 3.973 | 0.001*** |
| DN303 VS. XJ2 | 0.438 | 0.029* | 0.413 | 0.016* | 2.359 | 0.024* |
| DN303 VS. ZS5 | 0.478 | 0.023* | 0.296 | 0.031* | 2.633 | 0.01** |
| DN303 VS. ZS4 | 0.401 | 0.016* | 0.416 | 0.016* | 2.298 | 0.02* |
| E14 VS. HC1 | 0.433 | 0.029* | 0.257 | 0.030* | 2.126 | 0.039* |
| E14 VS. HE1 | 0.418 | 0.026* | 0.269 | 0.022* | 2.289 | 0.026* |
| E14 VS. HS4 | 0.392 | 0.01** | 0.291 | 0.006** | 2.216 | 0.017* |
| E14 VS. XJ2 | 0.438 | 0.008** | 0.335 | 0.009** | 2.446 | 0.007** |
| E14 VS. ZS5 | 0.475 | 0.065 | 0.155 | 0.105 | 1.726 | 0.06 |
| E14 VS. ZS4 | 0.404 | 0.056 | 0.263 | 0.055 | 2.05 | 0.049 |
| HC1 VS. HE1 | 0.403 | 0.032* | 0.278 | 0.043 | 2.507 | 0.041 |
| HC1 VS. HS4 | 0.377 | 0.002** | 0.365 | 0.005 | 2.632 | 0.003 |
| HC1 VS. XJ2 | 0.423 | 0.008** | 0.476 | 0.007 | 3.014 | 0.006 |
| HC1 VS. ZS5 | 0.458 | 0.087 | 0.181 | 0.074 | 1.838 | 0.065 |
| HC1 VS. ZS4 | 0.389 | 0.012* | 0.494 | 0.009** | 3.749 | 0.007** |
| HE1 VS. HS4 | 0.362 | 0.036* | 0.278 | 0.027* | 1.787 | 0.05 |
| HE1 VS. XJ2 | 0.408 | 0.017* | 0.369 | 0.018* | 2.678 | 0.014* |
| HE1 VS. ZS5 | 0.442 | 0.668 | -0.003 | 0.42 | 0.856 | 0.562 |
| HE1 VS. ZS4 | 0.373 | 0.005** | 0.5 | 0.009** | 4.66 | 0.007** |
| HS4 VS. XJ2 | 0.382 | 0.004** | 0.53 | 0.004** | 3.57 | 0.004** |
| HS4 VS. ZS5 | 0.414 | 0.11 | 0.232 | 0.033* | 1.453 | 0.084 |
| HS4 VS. ZS4 | 0.348 | 0.004** | 0.774 | 0.004** | 5.981 | 0.002** |
| XJ2 VS. ZS5 | 0.464 | 0.026* | 0.277 | 0.038* | 2.464 | 0.014* |
| XJ2 VS. ZS4 | 0.394 | 0.001*** | 0.571 | 0.006** | 3.86 | 0.004** |
| ZS5 VS. ZS4 | 0.426 | 0.011* | 0.509 | 0.005** | 3.988 | 0.009** |

Table S2 The dissimilarity test of fungal community structure based on Bray-Curtis distance.

| Dissimilarity result of Bray-Curtis distance | MRPP | | ANOSIM | | PERMANOVA | |
| --- | --- | --- | --- | --- | --- | --- |
|  | Delta | *P* | R | *P* | R^2^ | *P* |
| DN303 VS. E14 | 0.352 | 0.004** | 0.845 | 0.006** | 17.37 | 0.005** |
| DN303 VS. HC1 | 0.559 | 0.025* | 0.213 | 0.07 | 2.086 | 0.056 |
| DN303 VS. HE1 | 0.535 | 0.007** | 0.707 | 0.009** | 5.537 | 0.007** |
| DN303 VS. HS4 | 0.438 | 0.005** | 0.659 | 0.006** | 7.306 | 0.007** |
| DN303 VS. XJ2 | 0.406 | 0.008** | 0.707 | 0.004** | 4.841 | 0.001*** |
| DN303 VS. ZS5 | 0.571 | 0.013* | 0.532 | 0.013* | 4.147 | 0.016* |
| DN303 VS. ZS4 | 0.287 | 0.005** | 0.971 | 0.003** | 27.705 | 0.003** |
| E14 VS. HC1 | 0.439 | 0.003** | 0.419 | 0.002** | 5.877 | 0.003** |
| E14 VS. HE1 | 0.417 | 0.01** | 0.335 | 0.008** | 4.338 | 0.01** |
| E14 VS. HS4 | 0.328 | 0.041* | 0.089 | 0.107 | 2.799 | 0.016* |
| E14 VS. XJ2 | 0.299 | 0.003** | 1 | 0.003** | 40.03 | 0.005** |
| E14 VS. ZS5 | 0.439 | 0.026* | 0.299 | 0.013* | 2.735 | 0.009** |
| E14 VS. ZS4 | 0.19 | 0.003** | 0.796 | 0.005** | 16.334 | 0.003** |
| HC1 VS. HE1 | 0.607 | 0.025* | 0.247 | 0.015* | 2.303 | 0.023* |
| HC1 VS. HS4 | 0.518 | 0.032* | 0.193 | 0.045* | 2.51 | 0.037* |
| HC1 VS. XJ2 | 0.489 | 0.002** | 0.615 | 0.004** | 5.506 | 0.001*** |
| HC1 VS. ZS5 | 0.646 | 0.064 | 0.149 | 0.074 | 1.814 | 0.068 |
| HC1 VS. ZS4 | 0.38 | 0.003** | 0.615 | 0.003** | 9.201 | 0.005** |
| HE1 VS. HS4 | 0.495 | 0.067 | 0.139 | 0.068 | 1.962 | 0.071 |
| HE1 VS. XJ2 | 0.467 | 0.003** | 0.904 | 0.001*** | 10.997 | 0.006** |
| HE1 VS. ZS5 | 0.622 | 0.355 | 0.068 | 0.182 | 1.202 | 0.322 |
| HE1 VS. ZS4 | 0.357 | 0.01** | 0.454 | 0.01** | 6.832 | 0.009** |
| HS4 VS. XJ2 | 0.378 | 0.003** | 0.946 | 0.002** | 17.303 | 0.003** |
| HS4 VS. ZS5 | 0.525 | 0.158 | 0.131 | 0.085 | 1.377 | 0.208 |
| HS4 VS. ZS4 | 0.269 | 0.002** | 0.724 | 0.002** | 13.113 | 0.004** |
| XJ2 VS. ZS5 | 0.494 | 0.005** | 0.675 | 0.004** | 6.069 | 0.003** |
| XJ2 VS. ZS4 | 0.24 | 0.002** | 1 | 0.004** | 60.95 | 0.003** |
| ZS5 VS. ZS4 | 0.374 | 0.003** | 0.616 | 0.002** | 6.036 | 0.002** |

Table S3 Bacterial community topological properties of the empirical ecological networks at different cultivars in comparison to the random networks.

|  | **Empirical networks** | | | | | | | | | **Random networks** | | | |
| --- | --- | --- | --- | --- | --- | --- | --- | --- | --- | --- | --- | --- | --- |
|  | **cutoff** | **Total nodes** | **Total links** | **R squared of power-law** | **Average degree (avgK)** | **Average clustering coefficient (avgCC)** | **Average path distance (GD)** | **Harmonic geodesic distance (HD)** | **Modularity (module numbers)** | **Average clustering coefficient (avgCC)** | **Average path distance (GD)** | **Harmonic geodesic distance (HD)** | **Modularity (M)** |
| **DN303** | 0.94 | 398 | 867 | 0.763 | 4.357 | 0.646 | 7.182 | 13.511 | 0.773(51) | 0.34±0.02 | 4.956±0.179 | 5.734±0.232 | 0.683±0.012 |
| **E14** | 0.94 | 453 | 1109 | 0.702 | 4.896 | 0.7 | 8.138 | 13.319 | 0.845(42) | 0.418±0.015 | 5.356±0.173 | 5.301±0.172 | 0.731±0.01 |
| **HC1** | 0.94 | 430 | 1159 | 0.794 | 5.391 | 0.651 | 9.916 | 9.74 | 0.779(46) | 0.405±0.018 | 4.894±0.194 | 5.326±0.173 | 0.697±0.013 |
| **HE1** | 0.94 | 550 | 2226 | 0.699 | 8.095 | 0.674 | 6.639 | 7.375 | 0.735(45) | 0.524±0.011 | 4.581±0.189 | 4.917±0.155 | 0.68±0.01 |
| **HS4** | 0.95 | 717 | 1511 | 0.717 | 4.215 | 0.619 | 13.564 | 17.336 | 0.896(81) | 0.42±0.012 | 6.455±0.197 | 7.817±0.222 | 0.807±0.007 |
| **XJ2** | 0.94 | 517 | 1300 | 0.771 | 5.029 | 0.689 | 8.775 | 11.148 | 0.828(47) | 0.444±0.015 | 5.143±0.128 | 5.443±0.131 | 0.73±0.008 |
| **ZS5** | 0.94 | 397 | 926 | 0.749 | 4.665 | 0.748 | 7.308 | 18.051 | 0.839(51) | 0.408±0.021 | 4.849±0.181 | 5.597±0.222 | 0.704±0.014 |
| **ZS4** | 0.94 | 535 | 1425 | 0.808 | 5.327 | 0.678 | 7.336 | 13.698 | 0.815(52) | 0.455±0.014 | 5.36±0.174 | 5.668±0.17 | 0.725±0.011 |

Table S4 Fungal community topological properties of the empirical ecological networks at different cultivars in comparison to the random networks.

|  | **Empirical networks** | | | | | | | | | **Random networks** | | | |
| --- | --- | --- | --- | --- | --- | --- | --- | --- | --- | --- | --- | --- | --- |
|  | **cutoff** | **Total nodes** | **Total links** | **R squared of power-law** | **Average degree (avgK)** | **Average clustering coefficient (avgCC)** | **Average path distance (GD)** | **Harmonic geodesic distance (HD)** | **Modularity (module numbers)** | **Average clustering coefficient (avgCC)** | **Average path distance (GD)** | **Harmonic geodesic distance (HD)** | **Modularity (M)** |
| **DN303** | 0.94 | 134 | 224 | 0.783 | 3.343 | 0.436 | 4.703 | 4.174 | 0.694(12) | 0.079±0.022 | 3.874±0.095 | 3.481±0.106 | 0.545±0.012 |
| **E14** | 0.95 | 59 | 102 | 0.72 | 3.458 | 0.586 | 3.374 | 6.732 | 0.695(8) | 0.115±0.037 | 3.221±0.129 | 2.781±0.123 | 0.459±0.014 |
| **HC1** | 0.95 | 77 | 98 | 0.758 | 2.545 | 0.562 | 5.85 | 8.226 | 0.823(14) | 0.034±0.027 | 4.505±0.187 | 4.071±0.220 | 0.619±0.018 |
| **HE1** | 0.92 | 80 | 130 | 0.85 | 3.325 | 0.506 | 5.265 | 3.645 | 0.654(8) | 0.076±0.027 | 3.554±0.114 | 2.993±0.090 | 0.504±0.015 |
| **HS4** | 0.9 | 89 | 159 | 0.698 | 3.573 | 0.451 | 4.91 | 4.07 | 0.693(10) | 0.068±0.021 | 3.570±0.100 | 3.050±0.096 | 0.497±0.015 |
| **XJ2** | 0.94 | 115 | 130 | 0.868 | 2.261 | 0.455 | 3.902 | 16.954 | 0.851(20) | 0.028±0.020 | 5.165±0.286 | 5.636±0.368 | 0.702±0.014 |
| **ZS5** | 0.9 | 65 | 183 | 0.603 | 5.631 | 0.568 | 3.086 | 2.862 | 0.461(7) | 0.216±0.030 | 2.691±0.074 | 2.207±0.060 | 0.320±0.015 |
| **ZS4** | 0.9 | 59 | 94 | 0.823 | 3.186 | 0.569 | 2.381 | 6.267 | 0.692(11) | 0.091±0.032 | 3.432±0.149 | 2.904±0.150 | 0.497±0.017 |

Table S5 Taxonomic classification of connectors and module hubs of the bacterial community.

| **Cultivar** | **ID** | **Classification** | **ModuleID** | **Phylum** | **Class** | **Order** | **Family** | **Genus** |
| --- | --- | --- | --- | --- | --- | --- | --- | --- |
| **DN303** | OTU_17206 | Connectors | 10 | Bacteroidetes | Sphingobacteriia | Sphingobacteriales | Chitinophagaceae | Unclassified |
|  | OTU_21581 | Module.hubs | 4 | Proteobacteria | Gammaproteobacteria | Unclassified | Unclassified | Unclassified |
|  | OTU_28425 | Module.hubs | 2 | Actinobacteria | Actinobacteria | Actinomycetales | Mycobacteriaceae | Mycobacterium |
| **E14** | OTU_17719 | Connectors | 5 | Acidobacteria | Acidobacteria Gp1 | Unclassified | Unclassified | Acidobacterium |
|  | OTU_881 | Module.hubs | 21 | Actinobacteria | Actinobacteria | Acidimicrobiales | Acidimicrobineae | Aciditerrimonas |
|  | OTU_4980 | Module.hubs | 4 | Proteobacteria | Gammaproteobacteria | Unclassified | Unclassified | Unclassified |
|  | OTU_22857 | Module.hubs | 9 | Actinobacteria | Actinobacteria | Actinomycetales | Unclassified | Unclassified |
|  | OTU_11861 | Module.hubs | 6 | Actinobacteria | Actinobacteria | Solirubrobacterales | Conexibacteraceae | Conexibacter |
| **HC1** | OTU_11572 | Connectors | 31 | Proteobacteria | Alphaproteobacteria | Rhodospirillales | Acetobacteraceae | Unclassified |
|  | OTU_395 | Connectors | 2 | Planctomycetes | Planctomycetia | Planctomycetales | Planctomycetaceae | Aquisphaera |
|  | OTU_120 | Module.hubs | 5 | WPS-1 | Unclassified | Unclassified | Unclassified | WPS-1 |
|  | OTU_26538 | Module.hubs | 5 | Actinobacteria | Actinobacteria | Acidimicrobiales | Unclassified | Unclassified |
| **HE1** | OTU_11475 | Connectors | 19 | Acidobacteria | Acidobacteria Gp1 | Unclassified | Unclassified | Acidobacterium |
|  | OTU_8729 | Connectors | 4 | Acidobacteria | Acidobacteria Gp1 | Unclassified | Unclassified | Acidobacterium |
|  | OTU_9517 | Connectors | 36 | Acidobacteria | Acidobacteria Gp1 | Unclassified | Unclassified | Acidobacterium |
|  | OTU_28425 | Connectors | 6 | Actinobacteria | Actinobacteria | Actinomycetales | Mycobacteriaceae | Mycobacterium |
|  | OTU_7122 | Connectors | 3 | Proteobacteria | Alphaproteobacteria | Rhodospirillales | Rhodospirillaceae | Unclassified |
|  | OTU_6089 | Module.hubs | 9 | Proteobacteria | Alphaproteobacteria | Rhodospirillales | Acetobacteraceae | Acidisoma |
|  | OTU_6353 | Module.hubs | 3 | Verrucomicrobia | Subdivision3 | Unclassified | Unclassified | Subdivision3 |
|  | OTU_99 | Module.hubs | 36 | Actinobacteria | Actinobacteria | Actinomycetales | Dermacoccaceae | Barrientosiimonas |
| **HS4** | OTU_11833 | Module.hubs | 7 | Actinobacteria | Actinobacteria | Actinomycetales | Streptomycetaceae | Streptomyces |
|  | OTU_453 | Module.hubs | 3 | Bacteroidetes | Sphingobacteriia | Sphingobacteriales | Chitinophagaceae | Flavisolibacter |
|  | OTU_456 | Module.hubs | 52 | Acidobacteria | Acidobacteria Gp16 | Unclassified | Unclassified | Gp16 |
| **XJ2** | OTU_201 | Module.hubs | 4 | Proteobacteria | Alphaproteobacteria | Alphaproteobacteria | Unclassified | Rhizomicrobium |
|  | OTU_1209 | Module.hubs | 3 | Firmicutes | Bacilli | Bacillales | Thermoactinomycetaceae | Thermoactinomyces |
|  | OTU_819 | Module.hubs | 10 | Unclassified | Unclassified | Unclassified | Unclassified | Unclassified |
|  | OTU_541 | Module.hubs | 10 | Firmicutes | Bacilli | Bacillales | Bacillaceae 1 | Bacillus |
|  | OTU_29206 | Module.hubs | 11 | Bacteroidetes | Flavobacteriia | Flavobacteriales | Flavobacteriaceae | Flavobacterium |
| **ZS5** | OTU_5869 | Connectors | 25 | Bacteroidetes | Sphingobacteriia | Sphingobacteriales | Chitinophagaceae | Unclassified |
|  | OTU_23323 | Connectors | 5 | Proteobacteria | Alphaproteobacteria | Rhodospirillales | Acetobacteraceae | Acidicaldus |
|  | OTU_11572 | Module.hubs | 6 | Proteobacteria | Alphaproteobacteria | Rhodospirillales | Acetobacteraceae | Unclassified |
|  | OTU_391 | Module.hubs | 1 | Proteobacteria | Alphaproteobacteria | Unclassified | Unclassified | Unclassified |
| **ZS4** | OTU_17753 | Connectors | 4 | Proteobacteria | Gammaproteobacteria | Xanthomonadales | Sinobacteraceae | Alkanibacter |
|  | OTU_10361 | Connectors | 8 | Proteobacteria | Alphaproteobacteria | Rhodospirillales | Acetobacteraceae | Acidisoma |
|  | OTU_12950 | Module.hubs | 4 | Actinobacteria | Actinobacteria | Acidimicrobiales | Acidimicrobiaceae | Ferrimicrobium |
|  | OTU_22382 | Module.hubs | 4 | Acidobacteria | Acidobacteria Gp1 | Unclassified | Unclassified | Acidobacterium |
|  | OTU_27958 | Module.hubs | 22 | Proteobacteria | Gammaproteobacteria | Unclassified | Unclassified | Unclassified |
|  | OTU_8023 | Module.hubs | 8 | Proteobacteria | Gammaproteobacteria | Pseudomonadales | Unclassified | Unclassified |
|  | OTU_7602 | Module.hubs | 10 | Actinobacteria | Actinobacteria | Actinomycetales | Geodermatophilaceae | Unclassified |
|  | OTU_23194 | Module.hubs | 2 | Firmicutes | Bacilli | Bacillales | Planococcaceae | Lysinibacillus |

Table S6 Taxonomic classification of connectors and module hubs of the fungal community.

| Cultivar | ID | Classification | ModuleID | Phylum | Class | Order | Family | Genus |
| --- | --- | --- | --- | --- | --- | --- | --- | --- |
| DN303 | OTU_44 | Connectors | 6 | Ascomycota | Sordariomycetes | Unclassified | Unclassified | Unclassified |
|  | OTU_408 | Module.hubs | 2 | Zygomycota | Mucoromycotina Incertae sedis | Mucorales | Cunninghamellaceae | Gongronella |
|  | OTU_483 | Module.hubs | 7 | Ascomycota | Eurotiomycetes | Eurotiales | Trichocomaceae | Penicillium |
|  | OTU_1297 | Module.hubs | 5 | Ascomycota | Sordariomycetes | Sordariomycetidae Incertae sedis | Plectosphaerellaceae | Unclassified |
|  | OTU_3246 | Module.hubs | 3 | Ascomycota | Eurotiomycetes | Eurotiales | Trichocomaceae | Aspergillus |
| E14 |  |  |  |  |  |  |  |  |
| HC1 | OTU_2033 | Connectors | 7 | Ascomycota | Sordariomycetes | Sordariales | Chaetomiaceae | Humicola |
| HE1 | OTU_28 | Connectors | 4 | Ascomycota | Eurotiomycetes | Eurotiales | Trichocomaceae | Unclassified |
| HS4 | OTU_2120 | Connectors | 9 | Basidiomycota | Agaricomycetes | Unclassified | Unclassified | Unclassified |
|  | OTU_4701 | Connectors | 2 | Ascomycota | Eurotiomycetes | Eurotiales | Trichocomaceae | Unclassified |
|  | OTU_4917 | Connectors | 4 | Basidiomycota | Agaricomycetes | Trechisporales | Hydnodontaceae | Trechispora |
|  | OTU_192 | Module.hubs | 1 | Ascomycota | Pezizomycotina Incertae sedis | Pezizomycotina Incertae sedis | Pezizomycotina Incertae sedis | Ochroconis |
|  | OTU_208 | Module.hubs | 9 | Ascomycota | Sordariomycetes | Unclassified | Unclassified | Unclassified |
|  | OTU_3078 | Module.hubs | 3 | Ascomycota | Sordariomycetes | Sordariales | Chaetomiaceae | Thielavia |
| XJ2 | OTU_50 | Module.hubs | 1 | Ascomycota | Unclassified | Unclassified | Unclassified | Unclassified |
| ZS5 | OTU_9 | Connectors | 4 | Ascomycota | Eurotiomycetes | Eurotiales | Trichocomaceae | Eupenicillium |
|  | OTU_32 | Connectors | 2 | Ascomycota | Sordariomycetes | Sordariales | Sordariaceae | Neurospora |
| ZS4 |  |  |  |  |  |  |  |  |

Table S7 Network topological structure properties for the bacteria-fungi bipartite networks of the eight cultivars.

| **Cultivars** | | **DN303** | **E14** | **HC1** | **HE1** | **HS4** | **XJ2** | **ZS5** | **ZS4** |
| --- | --- | --- | --- | --- | --- | --- | --- | --- | --- |
| **Empirical network** | Total nodes | 173 | 128 | 163 | 153 | 125 | 169 | 128 | 98 |
|  | Total link | 502 | 238 | 383 | 370 | 190 | 493 | 287 | 169 |
|  | No.Bacteria | 121 | 98 | 111 | 123 | 89 | 121 | 98 | 79 |
|  | No.fungi | 52 | 30 | 52 | 30 | 36 | 48 | 30 | 19 |
|  | Positive percentage | 248 | 107 | 177 | 141 | 78 | 248 | 108 | 59 |
|  | Negative Link | 254 | 131 | 206 | 229 | 112 | 245 | 179 | 110 |
|  | Connectance | 0.08 | 0.081 | 0.066 | 0.1 | 0.059 | 0.085 | 0.098 | 0.113 |
|  | Web asymmetry | -0.399 | -0.531 | -0.362 | -0.608 | -0.424 | -0.432 | -0.531 | -0.612 |
|  | Links per species | 2.902 | 1.859 | 2.35 | 2.418 | 1.52 | 2.917 | 2.242 | 1.724 |
|  | Shannon diversity | 6.219 | 5.472 | 5.948 | 5.914 | 5.247 | 6.201 | 5.659 | 5.13 |
|  | nestedness | 16.613 | 13.816 | 8.003 | 13.784 | 8.095 | 17.514 | 13.868 | 11.599 |
|  | cluster coefficient | 0.066 | 0.071 | 0.045 | 0.065 | 0.056 | 0.074 | 0.066 | 0.063 |
|  | Specialization asymmetry | 0.106 | 0.291 | 0.047 | 0.264 | 0.237 | 0.209 | 0.23 | 0.32 |
|  | Modularity | 0.462 | 0.634 | 0.567 | 0.446 | 0.709 | 0.56 | 0.539 | 0.517 |
|  | No.of keystone | 21 | 11 | 8 | 17 | 6 | 12 | 9 | 9 |
|  | C.score.fungi | 0.874 | 0.868 | 0.87 | 0.849 | 0.904 | 0.86 | 0.846 | 0.807 |
|  | C.score.bacteria | 0.842 | 0.856 | 0.791 | 0.79 | 0.892 | 0.837 | 0.784 | 0.76 |
|  | functional.complementarity.fungi | 153.478 | 84.418 | 132.811 | 102.6 | 83.041 | 146.58 | 85.577 | 53.957 |
|  | functional.complementarity.bacteria | 192.138 | 88.834 | 144.947 | 118.193 | 83.713 | 178.41 | 89.884 | 53.483 |
| **Random network** | C.score.HL | 0.837±0.005 | 0.842±0.005 | 0.865±0.004 | 0.772±0.011 | 0.897±0.004 | 0.828±0.004 | 0.802±0.01 | 0.747±0.016 |
|  | C.score.LL | 0.801±0.004 | 0.839±0.003 | 0.759±0.005 | 0.73±0.005 | 0.882±0.002 | 0.805±0.003 | 0.734±0.006 | 0.721±0.006 |
|  | modularity(fast_greedy) | 0.35±0.007 | 0.474±0.009 | 0.4±0.008 | 0.367±0.006 | 0.563±0.011 | 0.35±0.007 | 0.388±0.008 | 0.454±0.009 |
|  | nestedness | 15.642±0.668 | 16.631±1.13 | 11.283±0.725 | 12.609±0.786 | 10.944±0.791 | 16.758±0.726 | 13.189±1.046 | 13.604±1.114 |
|  | specialisation asymmetry | 0.105±0.000 | 0.291±0.000 | 0.049±0.001 | 0.268±0.001 | 0.235±0.001 | 0.211±0.000 | 0.234±0.001 | 0.323±0.003 |

Table S8 Taxonomic classification of connectors and module hubs of the bipartite communities.

| **Cultivar** | **ID** | **Classification** | **Module ID** | **Domain** | **Phylum** | **Class** | **Order** | **Family** | **Genus** |
| --- | --- | --- | --- | --- | --- | --- | --- | --- | --- |
|  |  |  |  |  |  |  |  |  |  |
| **DN303** | BOTU_1 | Connector hubs | 8 | Bacteria | Proteobacteria | Gammaproteobacteria | Xanthomonadales | Xanthomonadaceae | Unclassified |
|  | BOTU_13 | Connector hubs | 10 | Bacteria | WPS-2 | Unclassified | Unclassified | Unclassified | WPS-2_genera_incertae_sedis |
|  | BOTU_14 | Connector hubs | 8 | Bacteria | Firmicutes | Bacilli | Bacillales | Planococcaceae | Unclassified |
|  | BOTU_153 | Connector hubs | 9 | Bacteria | Proteobacteria | Betaproteobacteria | Burkholderiales | Alcaligenaceae | Castellaniella |
|  | BOTU_161 | Connector hubs | 7 | Bacteria | Actinobacteria | Actinobacteria | Actinomycetales | Cryptosporangiaceae | Jatrophihabitans |
|  | BOTU_19694 | Connector hubs | 8 | Bacteria | Bacteroidetes | Sphingobacteriia | Sphingobacteriales | Chitinophagaceae | Unclassified |
|  | BOTU_38 | Connector hubs | 6 | Bacteria | Acidobacteria | Acidobacteria_Gp14 | Unclassified | Unclassified | Gp14 |
|  | BOTU_4636 | Connector hubs | 5 | Bacteria | Proteobacteria | Gammaproteobacteria | Xanthomonadales | Xanthomonadaceae | Luteibacter |
|  | BOTU_48 | Connector hubs | 8 | Bacteria | Proteobacteria | Gammaproteobacteria | Unclassified | Unclassified | Unclassified |
|  | BOTU_51 | Connector hubs | 7 | Bacteria | Proteobacteria | Alphaproteobacteria | Rhodospirillales | Acetobacteraceae | Unclassified |
|  | BOTU_62 | Connector hubs | 7 | Bacteria | Proteobacteria | Alphaproteobacteria | Rhodospirillales | Acetobacteraceae | Acidiphilium |
|  | BOTU_783 | Connector hubs | 7 | Bacteria | Actinobacteria | Actinobacteria | Actinomycetales | Nakamurellaceae | Nakamurella |
|  | FOTU_15 | Module hubs | 15 | Fungi | Basidiomycota | Agaricomycetes | Unclassified | Unclassified | Unclassified |
|  | FOTU_225 | Connector hubs | 19 | Fungi | Ascomycota | Eurotiomycetes | Eurotiales | Trichocomaceae | Unclassified |
|  | FOTU_28 | Connector hubs | 22 | Fungi | Ascomycota | Eurotiomycetes | Eurotiales | Trichocomaceae | Unclassified |
|  | FOTU_32 | Module hubs | 10 | Fungi | Ascomycota | Sordariomycetes | Sordariales | Sordariaceae | Neurospora |
|  | FOTU_3379 | Module hubs | 22 | Fungi | Ascomycota | Eurotiomycetes | Eurotiales | Trichocomaceae | Eupenicillium |
|  | FOTU_346 | Connector hubs | 9 | Fungi | Ascomycota | Eurotiomycetes | Eurotiales | Trichocomaceae | Phialosimplex |
|  | FOTU_41 | Network hubs | 36 | Fungi | Ascomycota | Unclassified | Unclassified | Unclassified | Unclassified |
|  | FOTU_6 | Module hubs | 14 | Fungi | Ascomycota | Sordariomycetes | Hypocreales | Hypocreaceae | Hypocrea |
|  | FOTU_82 | Module hubs | 20 | Fungi | Ascomycota | Unclassified | Unclassified | Unclassified | Unclassified |
| **E14** | BOTU_10262 | Connector hubs | 2 | Bacteria | Acidobacteria | Acidobacteria_Gp1 | Unclassified | Unclassified | Unclassified |
|  | BOTU_1047 | Connector hubs | 8 | Bacteria | Acidobacteria | Acidobacteria_Gp1 | Unclassified | Unclassified | Terriglobus |
|  | BOTU_18610 | Connector hubs | 2 | Bacteria | Proteobacteria | Gammaproteobacteria | Xanthomonadales | Xanthomonadaceae | Unclassified |
|  | BOTU_187 | Connector hubs | 2 | Bacteria | Actinobacteria | Actinobacteria | Actinomycetales | Microbacteriaceae | Gryllotalpicola |
|  | BOTU_36 | Connector hubs | 3 | Bacteria | Acidobacteria | Acidobacteria_Gp13 | Unclassified | Unclassified | Gp13 |
|  | FOTU_1 | Module hubs | 5 | Fungi | Basidiomycota | Agaricomycetes | Trechisporales | Hydnodontaceae | Trechispora |
|  | FOTU_15 | Module hubs | 7 | Fungi | Basidiomycota | Agaricomycetes | Unclassified | Unclassified | Unclassified |
|  | FOTU_2836 | Module hubs | 7 | Fungi | Basidiomycota | Unclassified | Unclassified | Unclassified | Unclassified |
|  | FOTU_4 | Connector hubs | 8 | Fungi | Ascomycota | Eurotiomycetes | Eurotiales | Trichocomaceae | Unclassified |
|  | FOTU_41 | Module hubs | 3 | Fungi | Ascomycota | Unclassified | Unclassified | Unclassified | Unclassified |
|  | FOTU_54 | Module hubs | 2 | Fungi | Ascomycota | Eurotiomycetes | Eurotiales | Trichocomaceae | Unclassified |
| **HC1** | BOTU_1685 | Connector hubs | 4 | Bacteria | Proteobacteria | Gammaproteobacteria | Xanthomonadales | Xanthomonadaceae | Unclassified |
|  | BOTU_17 | Module hubs | 2 | Bacteria | Bacteroidetes | Sphingobacteriia | Sphingobacteriales | Chitinophagaceae | Unclassified |
|  | BOTU_27507 | Connector hubs | 4 | Bacteria | Proteobacteria | Gammaproteobacteria | Xanthomonadales | Xanthomonadaceae | Unclassified |
|  | BOTU_7671 | Connector hubs | 6 | Bacteria | Acidobacteria | Acidobacteria_Gp1 | Unclassified | Unclassified | Acidobacterium |
|  | BOTU_91 | Connector hubs | 3 | Bacteria | Unclassified | Unclassified | Unclassified | Unclassified | Unclassified |
|  | FOTU_15 | Module hubs | 5 | Fungi | Basidiomycota | Agaricomycetes | Unclassified | Unclassified | Unclassified |
|  | FOTU_198 | Module hubs | 4 | Fungi | Basidiomycota | Unclassified | Unclassified | Unclassified | Unclassified |
|  | FOTU_54 | Module hubs | 5 | Fungi | Ascomycota | Eurotiomycetes | Eurotiales | Trichocomaceae | Unclassified |
| **HE1** | BOTU_10214 | Connector hubs | 1 | Bacteria | Acidobacteria | Acidobacteria_Gp1 | Unclassified | Unclassified | Acidobacterium |
|  | BOTU_107 | Connector hubs | 2 | Bacteria | Actinobacteria | Actinobacteria | Gaiellales | Gaiellaceae | Gaiella |
|  | BOTU_120 | Connector hubs | 1 | Bacteria | candidate division WPS-1 | Unclassified | Unclassified | Unclassified | WPS-1_genera_incertae_sedis |
|  | BOTU_128 | Connector hubs | 3 | Bacteria | Actinobacteria | Actinobacteria | Gaiellales | Gaiellaceae | Gaiella |
|  | BOTU_23910 | Connector hubs | 1 | Bacteria | Acidobacteria | Acidobacteria_Gp1 | Unclassified | Unclassified | Acidobacterium |
|  | BOTU_26282 | Connector hubs | 1 | Bacteria | Acidobacteria | Acidobacteria_Gp13 | Unclassified | Unclassified | Gp13 |
|  | BOTU_26870 | Connector hubs | 1 | Bacteria | Bacteroidetes | Sphingobacteriia | Sphingobacteriales | Chitinophagaceae | Sediminibacterium |
|  | BOTU_59 | Connector hubs | 2 | Bacteria | Acidobacteria | Acidobacteria_Gp2 | Unclassified | Unclassified | Gp2 |
|  | FOTU_12 | Module hubs | 3 | Fungi | Ascomycota | Eurotiomycetes | Eurotiales | Trichocomaceae | Talaromyces |
|  | FOTU_13 | Module hubs | 2 | Fungi | Basidiomycota | Agaricomycetes | Unclassified | Unclassified | Unclassified |
|  | FOTU_1837 | Module hubs | 4 | Fungi | Ascomycota | Eurotiomycetes | Eurotiales | Trichocomaceae | Unclassified |
|  | FOTU_2 | Module hubs | 2 | Fungi | Ascomycota | Sordariomycetes | Sordariales | Chaetomiaceae | Humicola |
|  | FOTU_28 | Module hubs | 4 | Fungi | Ascomycota | Eurotiomycetes | Eurotiales | Trichocomaceae | Unclassified |
|  | FOTU_3843 | Module hubs | 1 | Fungi | Ascomycota | Sordariomycetes | Sordariales | Chaetomiaceae | Humicola |
|  | FOTU_4239 | Module hubs | 4 | Fungi | Ascomycota | Eurotiomycetes | Eurotiales | Trichocomaceae | Eupenicillium |
|  | FOTU_603 | Connector hubs | 3 | Fungi | Ascomycota | Eurotiomycetes | Eurotiales | Trichocomaceae | Unclassified |
|  | FOTU_8 | Module hubs | 2 | Fungi | Basidiomycota | Unclassified | Unclassified | Unclassified | Unclassified |
| **HS4** | BOTU_39 | Connector hubs | 4 | Bacteria | Proteobacteria | Alphaproteobacteria | Rhizobiales | Bradyrhizobiaceae | Bradyrhizobium |
|  | FOTU_12 | Module hubs | 5 | Fungi | Ascomycota | Eurotiomycetes | Eurotiales | Trichocomaceae | Talaromyces |
|  | FOTU_4766 | Module hubs | 3 | Fungi | Ascomycota | Eurotiomycetes | Eurotiales | Trichocomaceae | Eupenicillium |
|  | FOTU_54 | Module hubs | 1 | Fungi | Ascomycota | Eurotiomycetes | Eurotiales | Trichocomaceae | Unclassified |
|  | FOTU_57 | Module hubs | 7 | Fungi | Zygomycota | Mucoromycotina Incertae sedis | Mucorales | Umbelopsidaceae | Umbelopsis |
|  | FOTU_9 | Module hubs | 3 | Fungi | Ascomycota | Eurotiomycetes | Eurotiales | Trichocomaceae | Eupenicillium |
| **XJ2** | BOTU_104 | Connector hubs | 2 | Bacteria | Chloroflexi | Ktedonobacteria | Ktedonobacterales | Ktedonobacteraceae | Ktedonobacter |
|  | BOTU_17758 | Connector hubs | 2 | Bacteria | Acidobacteria | Acidobacteria_Gp1 | Unclassified | Unclassified | Acidobacterium |
|  | BOTU_27059 | Connector hubs | 2 | Bacteria | Proteobacteria | Gammaproteobacteria | Xanthomonadales | Xanthomonadaceae | Rhodanobacter |
|  | BOTU_783 | Connector hubs | 1 | Bacteria | Actinobacteria | Actinobacteria | Actinomycetales | Nakamurellaceae | Nakamurella |
|  | FOTU_13 | Module hubs | 2 | Fungi | Basidiomycota | Agaricomycetes | Unclassified | Unclassified | Unclassified |
|  | FOTU_41 | Connector hubs | 1 | Fungi | Ascomycota | Unclassified | Unclassified | Unclassified | Unclassified |
|  | FOTU_4766 | Module hubs | 3 | Fungi | Ascomycota | Eurotiomycetes | Eurotiales | Trichocomaceae | Eupenicillium |
|  | FOTU_48 | Module hubs | 5 | Fungi | Ascomycota | Eurotiomycetes | Eurotiales | Trichocomaceae | Unclassified |
|  | FOTU_57 | Module hubs | 6 | Fungi | Zygomycota | Mucoromycotina Incertae sedis | Mucorales | Umbelopsidaceae | Umbelopsis |
|  | FOTU_603 | Connector hubs | 2 | Fungi | Ascomycota | Eurotiomycetes | Eurotiales | Trichocomaceae | Unclassified |
|  | FOTU_7 | Module hubs | 3 | Fungi | Ascomycota | Sordariomycetes | Hypocreales | Nectriaceae | Gibberella |
|  | FOTU_8 | Module hubs | 2 | Fungi | Basidiomycota | Unclassified | Unclassified | Unclassified | Unclassified |
| **ZS5** | BOTU_51 | Connector hubs | 3 | Bacteria | Proteobacteria | Alphaproteobacteria | Rhodospirillales | Acetobacteraceae | Unclassified |
|  | BOTU_70 | Connector hubs | 3 | Bacteria | Proteobacteria | Alphaproteobacteria | Rhodospirillales | Acetobacteraceae | Unclassified |
|  | BOTU_94 | Connector hubs | 3 | Bacteria | Unclassified | Unclassified | Unclassified | Unclassified | Unclassified |
|  | FOTU_28 | Module hubs | 4 | Fungi | Ascomycota | Eurotiomycetes | Eurotiales | Trichocomaceae | Unclassified |
|  | FOTU_32 | Module hubs | 1 | Fungi | Ascomycota | Sordariomycetes | Sordariales | Sordariaceae | Neurospora |
|  | FOTU_41 | Module hubs | 2 | Fungi | Ascomycota | Unclassified | Unclassified | Unclassified | Unclassified |
|  | FOTU_4239 | Module hubs | 4 | Fungi | Ascomycota | Eurotiomycetes | Eurotiales | Trichocomaceae | Eupenicillium |
|  | FOTU_87 | Module hubs | 4 | Fungi | Unclassified | Unclassified | Unclassified | Unclassified | Unclassified |
|  | FOTU_9 | Module hubs | 3 | Fungi | Ascomycota | Eurotiomycetes | Eurotiales | Trichocomaceae | Eupenicillium |
| **ZS4** | BOTU_13 | Connector hubs | 2 | Bacteria | candidate division WPS-2 | Unclassified | Unclassified | Unclassified | WPS-2_genera_incertae_sedis |
|  | BOTU_2577 | Connector hubs | 4 | Bacteria | Proteobacteria | Alphaproteobacteria | Rhodospirillales | Acetobacteraceae | Acidisoma |
|  | BOTU_29 | Connector hubs | 4 | Bacteria | Proteobacteria | Alphaproteobacteria | Alphaproteobacteria_incertae_sedis | Unclassified | Rhizomicrobium |
|  | BOTU_42 | Connector hubs | 2 | Bacteria | Actinobacteria | Actinobacteria | Actinomycetales | Microbacteriaceae | Humibacter |
|  | FOTU_15 | Module hubs | 4 | Fungi | Basidiomycota | Agaricomycetes | Unclassified | Unclassified | Unclassified |
|  | FOTU_4239 | Module hubs | 3 | Fungi | Ascomycota | Eurotiomycetes | Eurotiales | Trichocomaceae | Eupenicillium |
|  | FOTU_4766 | Module hubs | 3 | Fungi | Ascomycota | Eurotiomycetes | Eurotiales | Trichocomaceae | Eupenicillium |
|  | FOTU_87 | Module hubs | 2 | Fungi | Unclassified | Unclassified | Unclassified | Unclassified | Unclassified |
|  | FOTU_9 | Module hubs | 1 | Fungi | Ascomycota | Eurotiomycetes | Eurotiales | Trichocomaceae | Eupenicillium |
